# The Relation Between Psychopathy and Sexual Aggression: A Meta‐Analysis

**DOI:** 10.1111/jopy.70017

**Published:** 2025-08-31

**Authors:** Melissa Packer West, Tianwei V. Du, Kaela Van Til, Joshua D. Miller, Donald R. Lynam

**Affiliations:** ^1^ Department of Psychological Sciences Purdue University West Lafayette Indiana USA; ^2^ Department of Psychiatry and Behavioral Sciences Stanford University School of Medicine Stanford California USA; ^3^ Department of Psychology University of Georgia Athens Georgia USA

**Keywords:** meta‐analysis, personality, psychopathy, sexual aggression, triarchic model of psychopathy

## Abstract

**Objective:**

Psychopathy is a multifaceted, hierarchical construct that has been linked to aggression and antisocial behavior. The triarchic model of psychopathy comprises three underlying, distinct trait domains: boldness, disinhibition, and meanness. Understanding how psychopathy at general and factor levels relates to sexual aggression is critical given its connection and the serious repercussions of sexual aggression.

**Method:**

This preregistered meta‐analysis (*k* = 117) examined the relationship between psychopathy at the total construct and triarchic levels and sexual aggression in adult samples. A relative weights analysis was used to produce triarchic psychopathy scores from commonly used psychopathy measures and the Triarchic Psychopathy Measure (TriPM).

**Results:**

Psychopathy evinced a moderate, positive relationship with general sexual aggression as well as most specific forms of sexual aggression except for rape and child molestation. At the triarchic level, meanness and disinhibition related positively and boldness negatively to sexual aggression. Other moderation analyses revealed significant heterogeneity in study methods and characteristics that can explain variability in psychopathy's relations with sexual aggression.

**Conclusions:**

Psychopathy relates moderately to various forms of sexual aggression. The relationship depends on multiple factors. Understanding these mechanisms can inform prevention, treatment, and risk assessment of damaging sexual acts.

Psychopathy is a personality construct associated with dysfunction in several domains, including perspective‐taking (e.g., Brown et al. 2015), aggression (e.g., Walters 2003), and substance overuse (e.g., Lynam et al. 2013). Cleckley (1941) defined psychopathy by the “mask of sanity” which hid serious underlying pathology. Psychopathy is not an official diagnosis included in the fifth edition of the Diagnostic and Statistical Manual of Mental Disorders (DSM‐5; American Psychiatric Association 2013), largely due to a lack of consensus on its conceptualization (e.g., Crego and Widiger 2015), but the Alternative Model of Personality Disorders Section III of the DSM‐5 includes it as a specifier for the diagnosis of antisocial personality disorder.

The development of the Psychopathy Checklist and its revision (PCL, PCL‐R; Hare 1980, 1991, 2003), which uses an extensive file review and interview, afforded a useful assessment that jump‐started research on psychopathy (Hare and Neumann 2008). The PCL measures focus on traits such as grandiosity, impulsivity, irresponsibility, superficial charm, manipulativeness, callousness, and shallow affect. Stemming from a need to measure psychopathy outside of forensic settings, several self‐report assessments have been developed, such as the Triarchic Psychopathy Measure (TriPM; Patrick 2010), Psychopathic Personality Inventory (PPI, Lilienfeld and Andrews 1996; Lilienfeld and Widows 2005), Levenson Self‐Report Psychopathy Scale (LSRP; Levenson et al. 1995), Self‐Report Psychopathy Scale (SRP; Paulhus et al. 2016), and Elemental Psychopathy Assessment (EPA; Lynam et al. 2011). These measures are multidimensional and assess similar dimensions. For example, using the Five‐Factor Model of personality as a framework for comparing psychopathy measures, LSRP Factor 1, SRP Interpersonal Manipulation, SRP Callous Affect, and TriPM Meanness have been found to measure very similar dimensions because they comprise mostly low agreeableness (Lynam and Miller 2015). Subscales from other measures, including SRP Erratic Lifestyle, SRP Antisocial Behavior, PPI Self‐Centered Impulsivity, TriPM Disinhibition, and LSRP Factor 2, comprise primarily low conscientiousness, low agreeableness, and high externalizing‐based neuroticism (e.g., angry hostility; Lynam and Miller 2015). Some measures of psychopathy also contain a factor related to social assertiveness, fearlessness, and risk taking to describe certain features highlighted by Cleckley; these scales include PPI Fearless Dominance and TriPM Boldness that represent low Neuroticism and high Extraversion from an FFM perspective (Miller and Lynam 2012; Sleep et al. 2019).

In the present paper, we refer to these similar dimensions across psychopathy measures as meanness, disinhibition, and boldness, which make up the triarchic model of psychopathy (Patrick 2022; see Patrick et al. 2009 and Patrick and Drislane 2015 for empirical reviews and Sleep et al. 2019 for a meta‐analytic review), and focus on these dimensions in adults. Patrick and Drislane (2015) state that “Disinhibition entails impulsiveness, weak restraint, hostility and mistrust, and difficulties in regulating emotion. Meanness entails deficient empathy, lack of affiliative capacity, contempt toward others, predatory exploitativeness, and empowerment through cruelty or destructiveness. The third construct in the triarchic model, boldness, entails proclivities toward confidence and social assertiveness, emotional, and venturesomeness” (p. 628). The original, most commonly used measure to assess this model is the TriPM (Patrick et al. 2009) which renders a total psychopathy score and scores for the three domains. However, the triarchic psychopathy dimensions can be operationalized using items from various other psychopathy and personality measures (e.g., NEO PI‐R: Drislane et al. 2018; Psychopathic Personality Inventory: Hall et al. 2014). To be clear, while the triarchic model of psychopathy has become a prominent model of psychopathy, there are concerns surrounding the TriPM and other measures developed to index its constructs, including their factor structure (Collison et al. 2021; Roy et al. 2021), the distinguishability of their disinhibition and meanness scales (Sleep et al. 2019), the distinctiveness of the domains they assess from those of the Five Factor Model (Collison et al. 2023), and whether boldness should be considered a central component of psychopathy given weak relations of its scale operationalizations with disinhibition and meanness scales and their stronger associations with adaptive versus maladaptive outcomes (e.g., Sleep et al. 2019; for a conceptually based discussion of the latter issue, see Crego and Widiger 2015). The choice to use the triarchic model as an organizing framework was practical: it represents common dimensions of psychopathy and there are sufficient data on it to use the current analytic approach.

Sexual aggression (SA) is defined as any method used to obtain some kind of sexual activity from an individual who is unwilling or not able to consent (e.g., Abbey et al. 2007). It can involve coercion in the form of threats or use of physical force, abuse of a position of power, capitalizing on a victim's inability to resist, and verbal manipulation (Krahé 2020). There are several risk factors for committing SA, including childhood sexual, emotional, and physical abuse (Abbey et al. 2006; Peterson et al. 2018), negative affect (i.e., anger, anxiety, depression; Peterson et al. 2018), hostile masculinity, psychopathy (e.g., Brazil et al. 2023), impersonal sex (Abbey et al. 2011), a tendency to misinterpret women's sexual cues (Abbey et al. 2011), delinquency (Abbey et al. 2012), alcohol consumption (Abbey et al. 2011), hypersexuality, sexual preoccupation, and sexual compulsivity (Knight and Graham 2017).

Some previous research has indicated strong associations between psychopathy and SA (Knight and Guay 2006, 2018). For example, psychopathic criminals evince more sexually coercive behavior than non‐psychopathic criminals (e.g., DeGue et al. 2010) and psychopathy is putatively a salient feature in particularly violent sexual offenders (e.g., Porter et al. 2003). Psychopathy and SA account for significant societal issues (Marshall 1992; Prentky and Burgess 1990), such as costs related to incarceration, victim and perpetrator treatment, investigations, and judicial processes as well as harm to victims, families, and communities. Psychopathic sexual offenders are more likely to recidivate (Hawes et al. 2013) and are generally more difficult to treat than non‐psychopathic offenders (e.g., Barbaree et al. 2006). Their resistance to treatment and disruptive behavior during interventions pose significant challenges (Hildebrand et al. 2004). However, treatment advancements, including the Risk‐Need‐Responsivity (RNR) treatment approach, which frames psychopathy as a responsivity issue rather than a contraindication for treatment, show promise (e.g., Bonta and Andrews 2023; Olver and Riemer 2021).

Previous meta‐analyses on SA and psychopathy have focused on psychopathy as a predictor of sexual recidivism, but no meta‐analysis to date has examined the basic association between SA and psychopathy. There also is no existing meta‐analysis that has investigated the relationship between SA and the separable aspects of psychopathy nor between multiple different forms of SA (e.g., child molestation, rape) and psychopathy. This lack of research hamstrings the ability to determine whether the relation between psychopathy and sexually aggressive conduct is the same across all dimensions of psychopathy. Accordingly, the aim of the current study is to conduct a meta‐analysis to comprehensively inspect the association between psychopathy at the global and triarchic levels and SA in general and in specific forms.

Although formally meta‐analyzing how the triarchic model of psychopathy relates to SA is not possible directly given the relative lack of studies specifically examining these relations, it is feasible to infer the relation by capitalizing on known associations between the triarchic psychopathy factors and commonly used psychopathy measures using a relative weights analysis (see Du et al. (2022) for an example; Tonidandel and LeBreton 2015). A relative weights analysis fully partitions the variance accounted for in an outcome variable by predictors; the resultant percentages denote how much boldness, meanness, and disinhibition are contained in a given psychopathy measure and can be used to estimate how effect sizes of psychopathy on SA vary according to these relative weights across various measures of psychopathy. Relative weights of these three factors based on the TriPM in frequently used psychopathy measures (e.g., PCL‐R, PPI, LSRP, SRP, and EPA) can be obtained from correlation matrices from existing studies (e.g., TriPM and PCL‐R: Venables et al. 2014).

The current preregistered meta‐analysis comprises three parts: (1) a meta‐analysis of psychopathy's general association with SA among adults. If a study only contained subscale scores, we averaged those scores to produce a total score. To account for the contribution of multiple effect sizes from studies using multiple measures of psychopathy or of SA, we included study number as a random effect in our analysis; (2) individual meta‐analyses of general psychopathy's association with various SA forms measured by studies (e.g., rape, child molestation). The SA forms examined were those rendered by what studies measured as outcomes and those that produced *k* ≥ 3, a threshold used in a related meta‐analysis (Vize et al. 2018). To our knowledge, there is no established SA coding scheme; thus, we used common legal categories (e.g., rape, child molestation) as a guide for identifying categories of SA forms to use for separate meta‐analyses); (3a) moderation analyses of how general psychopathy's association with general SA as well as with more specific forms of SA varies according to the percentage of each of the three trait domains of psychopathy in the psychopathy measure used; and (3b) moderation analyses of how general psychopathy's association with general SA as well as with more specific forms of SA that have *k* ≥ 20 effect sizes varies according to the following other moderators^1^: (a) type of sample used (e.g., forensic, community), (b) source of information for psychopathy and SA measure used, separately (e.g., self‐report), (c) mean age, (d) gender (coded by percentage male), (e) race (coded by percentage white), (f) sample's country of origin, (g) measure of psychopathy (e.g., PCL‐R, SRP), (h) measure of SA (e.g., Sexual Experiences Survey—Perpetration Form), (i) sexual offender sample type, when applicable, and (j) victim class of perpetrators (adult, child, mixed [both adults and children]). Because we anticipated that several moderators would be highly correlated with each other, we conducted separate moderation analyses for each of these variables. A moderation approach was used to study the association between psychopathy triarchic trait domains and SA because it would help maximize the number of studies that could be used in the analysis. It also allowed for the examination of how the effect of psychopathy on SA varies according to the measured percentages of lower‐order psychopathy factors contained in the measure used.

The following research questions were posed: (1) To what extent does general psychopathy correlate with SA?^2^ We expected that general psychopathy would associate positively and strongly with SA, as has been found in previous literature. (2) To what extent does general psychopathy correlate with more specific forms of SA? Based on previous literature, we expected that rape, as the SA form, would have a strong, positive association with psychopathy. Given that the normative score for child sexual offenders on the PCL‐R, the most widely used psychopathy measure used in forensic settings in relation to antisocial behavior, falls in between the normative scores for non‐psychopathic individuals and other types of sexual offenders (Hare 1991, 2003), we expected that child molestation would evince a positive, but weaker association with psychopathy than other forms of SA. (3) To what extent do boldness, meanness, disinhibition, and other moderators influence the relation between general psychopathy and SA? Based on the literature, we expected that higher percentages of meanness and disinhibition, and mixed as the victim class, would be associated with stronger, more positive associations between psychopathy and SA. We expected positive but weaker associations between psychopathy and SA for children as the victim class.

## 1 Method

### 1.1 Literature Screening

A comprehensive literature search consistent with the Preferred Reporting Items for Systematic Review and Meta‐Analysis guidelines (PRISMA; Moher et al. 2009) was conducted in PubMed and PsycINFO (which includes unpublished dissertations and theses). To identify studies that measure both psychopathy and SA, we used the following search terms: (Sex offen* OR mixed sex offen* OR sex crim* OR sexual violence OR sexual homicide OR homicidal sex offen* OR sexual aggress* OR sexual coercion OR sexual victimization OR sexual perpetration OR sexual devian* OR sexual abuse OR sexual coercion OR rape OR rapist OR unwanted sex OR forced sex OR sexual batter* OR sodomy OR sexual assault OR pedo* OR child sex offen* OR child molest* OR child sexual abuse OR child porn* OR incest OR paraphil* OR sexual sadism OR exhibition* OR indecent exposure OR sex traffick* OR internet sex crim* OR online sex crim* OR sex traffick* OR sexual intimate partner* OR dating sexual violence OR dating sexual aggression OR intimate sexual violence OR intimate sexual aggression OR partner sexual violence OR partner sexual aggression OR dating sexual abuse OR partner sexual abuse) AND (psychopathy OR psychopathic OR Psychopathy Checklist OR Psychopathy Checklist‐Revised OR Triarchic Psychopathy Measure OR Levenson Self‐Report Psychopathy Scale OR Self‐Report Psychopathy Scale OR Psychopathic Personality Inventory OR Interpersonal Measure of Psychopathy OR Minnesota Multiphasic Personality Inventory–2–Restructured Form Triarchic Psychopathy Scales OR Multidimensional Personality Questionnaire Triarchic Psychopathy Scales OR Elemental Psychopathy Assessment OR HEXACO‐Tri OR NEO Personality Inventory‐Revised Triarchic Scales).

We confined the results to studies that were published from January 1, 1980, to January 16, 2023, to align with the advent of the original Psychopathy Checklist and a modern, relatively cohesive conceptualization of psychopathy at the broad level. To be included in our analysis, studies needed to be in English, report empirical findings, include at least one measure of psychopathy and one measure of SA, and collect data from an adult sample. We excluded studies that did not meet any of the inclusion criteria or used only a behavioral task to measure psychopathy or SA. A study was permitted to report associations between either dimensional levels of psychopathy or a categorical diagnosis of psychopathy (e.g., scoring above the cut‐off on the PCL‐R) and SA. SA could be reported as a continuous frequency variable (e.g., number of acts in the past year), a lifetime yes/no count for any perpetration of a sexually aggressive act, a criminal record indicating a prior or current offense involving SA, and a treatment referral for SA perpetration. We only included adult sample studies because most psychopathy and SA measures have been developed and validated in adult populations. Studies reporting on more than one sample were treated as independent samples and included in the analysis separately. If the same sample was included in multiple studies, it was included only once in the meta‐analysis. This study and its analysis plan were preregistered in adherence to the requirements of the Center for Open Science: https://osf.io/u6jze/?view_only=3ab83176bffc47a68f3ee6d7c10706ea.

After reviewing the titles and abstracts, articles were excluded based on our a priori exclusion criteria. For search results without full‐text availability online, authors were contacted to solicit the full‐text article. To gather unpublished data as well, in addition to using PsycINFO (which includes unpublished dissertations and theses) as one of the research databases for our search, we requested unpublished data through several academic listservs. We also solicited specific researchers who were authors on at least two studies qualifying for our meta‐analysis for unpublished data. The remaining full texts were then examined for statistics needed to calculate effect sizes. When this information was not available, study authors were emailed for the information. Eight of 31 authors responded with the necessary effect sizes. We also searched through the references of our included studies and related meta‐analyses for additional studies that meet our criteria; four studies were added through this method.

### 1.2 Coding and Statistical Analyses

After ascertaining eligible studies, multiple variables were extracted and formally coded. We coded effect sizes (*r*s) between psychopathy and SA, pertinent demographic data (mean age, gender and race percentages), sample size, sample type (e.g., forensic, student), source of information for psychopathy and SA (self‐report, informant‐report, clinician ratings, interview, official records review), measure of psychopathy (e.g., LSRP, PCL‐R), measure of SA (e.g., criminal records), sexual offender sample type, as applicable, and victim class of perpetrators. To correct for the influence of measurement error, we also coded the reliability (e.g., Cronbach's alpha) of the psychopathy and SA measures to calculate disattenuated effect sizes. If reliability statistics were not provided by a study author, average reliabilities from the other studies using the same assessment were used to calculate disattenuated effects.^3^

For calculating the relation of psychopathy at the triarchic level and SA, we used a relative weights analysis (Tonidandel and LeBreton 2015) to acquire the percentage of lower‐order psychopathy factors represented in each of the commonly used psychopathy measures. This approach allowed for the inclusion of studies that did not directly measure the triarchic model of psychopathy. Relative weights analyses help determine how correlated predictors associate with an outcome variable by producing a set of new, orthogonal predictors that are maximally associated with original predictors. The outcome variable can then be regressed onto the new uncorrelated predictors to render standardized regression coefficients, which are rescaled to calculate the relative weights for the predictor variables. In sum, a relative weights analysis can be accomplished with correlation matrices of predictor and outcome variables. An informal preliminary review of the literature revealed that such matrices between the TriPM (the most commonly used measure of the triarchic model of psychopathy) and psychopathy measures that do not produce scores for the triarchic factors exist across studies and could be used for the current study's relative weights analysis. The relative weights rendered from the current analysis were used to estimate how effect sizes of psychopathy on SA vary according to these relative weights across various measures of psychopathy using a novel moderator analysis approach. We coded effects of boldness (*B*), meanness (*M*), and disinhibition (*D*) if a study reported them (only two studies did) and otherwise entered relative weights calculated for a given psychopathy measure (e.g., rescaled relative weights for SRP‐III and PCL‐R, respectively, were *B* = 21.56, 32.06; *M* = 44.63, 36.10; *D* = 33.82, 31.84).

The first author coded the above information for all articles independently. Roughly 25% of the articles were independently reviewed by the third author; any resultant discrepancies were resolved. Interrater reliability was calculated using Cohen's kappa and percentage of agreement for qualitative variables and intraclass coefficients (ICC) for continuous variables, revealing an average kappa of 0.91, percentage of agreement—95%, and ICC—0.97.

The analyses were conducted using the metafor package (Viechtbauer 2010) in RStudio (RStudio Team 2016), including its escalc, rma, funnel, and trimfill functions. Effect sizes were produced by converting reported effect sizes (e.g., Cohen's *d*, *t*‐tests, chi‐squared tests) to Pearson correlation coefficients (*r*) or calculated using reported sample sizes, means, and standard deviations and the practical meta‐analysis effect size calculator (Lipsey and Wilson 2001). We then produced mean effect sizes and their 95% confidence intervals for each relation of concern between psychopathy and SA. All effect sizes were transformed into *z*′ units using the Fisher *z*′ transformation (Lipsey and Wilson 2001) prior to being averaged; they were then transformed back to an unstandardized zero‐order *r* to yield a weighted mean effect size. To produce a mean effect size across study samples for each form of SA included in the current study, random‐effects models were run in R using the rma function in which each effect size (*ESi*) was weighted by the inverse of its variance (*wi*) via the Restricted Maximum Likelihood Estimator. For analysis at the triarchic level, relative weights of each psychopathy lower‐order factor were included in the analysis as moderators. For example, we examined how the relationship between psychopathy and SA changes as a function of the amount of meanness (as signified by relative weights) present in each psychopathy measure. Other moderation analyses, such as for victim type and sex offender sample type, were conducted. All moderation analyses were conducted using a meta‐regression approach.

#### 1.2.1 Heterogeneity in Effect Size, Outlier Identification, and Publication Bias

To assess overall heterogeneity that can be ascribed to true between‐studies variation, *Q* and *I* ^2^ statistics were calculated and reported (Higgins 2003). All effect sizes were transformed to *z*′ units; values of *z*′ not in the range of −2.5 to 2.5 standard deviations were deemed outliers (Du et al. 2022; Rousseeuw 1990). We report results both with and without the outliers. For each predictor, we used funnel plots to evaluate publication bias. This method enables the detection of potential bias due to underrepresentation of studies with small samples. We used the trim and fill method and step function selection models (Duval and Tweedie 2000; Hedges 1992; Vevea and Woods 2005) to adjust for any potential publication bias and to estimate and impute potentially missing studies. We also included study number in the models as a random effect to account for dependency produced by studies that reported multiple effects (e.g., studies that used multiple measures to assess psychopathy and/or SA) and more generally to account for variation that may come from the individual studies that made up our meta‐analysis.

#### 1.2.2 Relative Weights

To find correlation matrices used to calculate relative weights of boldness, meanness, and disinhibition represented in psychopathy measures used in studies included in the current meta‐analysis, we used the following process: We tried to find a correlation matrix in a published study for the psychopathy measure, ideally according to its specific version, in an adult sample of *N* = > 100. If a correlation matrix came from a sample smaller than 100 but used the specific version of a given psychopathy measure, we used it. If we could not find a correlation matrix using the specific measure version of a given psychopathy measure *and* the study sample was less than 100, we used the correlation matrix of a measure version most similar to it (e.g., PPI‐R for PPI‐40 since PPI‐40 is based off of PPI‐R). TriPM intercorrelations were used from the same study that provided correlations between TriPM and the measure in question if they were provided; otherwise, TriPM intercorrelations from Sharpe et al. (2021; *r*Total:B = 0.65, *r*Total:M = 0.84, *r*Total:D = 0.64; *r*B:M = 0.29, *r*B:D = −0.02; *r*M:D = 0.49) were used to calculate relative weights for a given psychopathy measure. If we could not find any correlation matrix between TriPM and the psychopathy measure in question, which ended up being the case for only two psychopathy measures in the current study (i.e., Dirty Dozen; Naughty Nine), that measure was not included in the triarchic‐level psychopathy moderation analysis.

## 2 Results

This meta‐analysis included 117 independent samples from 95 studies involving 41,009 participants. Among the 95 studies, 70 were published in peer‐reviewed journals, 21 were doctoral dissertations, and four were unpublished studies. The average age across the samples was 32.14 (SD = 8.23). On average, the samples were 85.68% male and 67.12% White. Of all 117 samples, 65 were forensic samples (35 of which comprised sexual offenders only), 24 were student samples, 16 were community samples, and 12 were a combination of forensic and non‐forensic samples. See Table S1 for characteristics of all studies included, Appendix A in Supporting Information for their full references, and Figure S1 for the PRISMA flowchart delineating the process of identifying, screening, and including/excluding potential studies for this meta‐analysis. See Table S2 for the relative weights calculated for each psychopathy measure used in the studies included in this meta‐analysis and Appendix B in Supporting Information for the references of the studies used to derive relative weights.

### 2.1 Global Psychopathy Effects

The random‐effects, meta‐analytically derived effect sizes for the relations between overall psychopathy and overall SA and more specific forms of SA are presented in Table 1. Psychopathy was significantly, positively associated with general SA (*r* = 0.30), exhibitionism (*r* = 0.15), undifferentiated SA (*r* = 0.37), voyeurism (*r* = 0.38), mixed SA (*r* = 0.20), sexual homicide (*r* = 0.28), sexual cyberbullying/harassment (*r* = 0.51), and sexual sadism (*r* = 0.33). Psychopathy was not significantly associated with rape (*r* = 0.18) or child molestation (*r* = −0.08).

**TABLE 1:** Meta‐analytically derived relations between sexual aggression and overall psychopathy.

	Overall psychopathy					
	*k*	*N*	SE	Weighted ES	95% CI	*τ*/*σ* ^a^
General sexual aggression						
Original	167	47,622	0.02	0.30***	0.26–0.35	0.28
With study number as random effect	167	47,622	0.03	0.28***	0.22–0.34	0.28
Trim‐and‐fill	172	47,904	0.02	0.33***	0.28–0.37	0.30
Selection models	167	47,622	0.05	0.32***	0.23–0.41	0.27
Outliers removed	161	46,804	0.02	0.28***	0.24–0.32	0.25
General sexual aggression no CM						
Original	147	46,305	0.02	0.35***	0.31–0.39	0.24
With study number as random effect	147	46,305	0.03	0.33***	0.27–0.38	0.25
Trim‐and‐fill	147	46,305	0.02	0.35***	0.31–0.39	0.24
Selection models	147	46,305	0.03	0.39***	0.32–0.45	0.23
Outliers removed	141	45,487	0.02	0.33***	0.29–0.37	0.21
Rape						
Original	8	762	0.12	0.18	−0.06–0.41	0.30
With study number as random effect	8	762	0.12	0.18	−0.06–0.41	0.30
Trim‐and‐fill	8	762	0.12	0.18	−0.06–0.41	0.30
Selection models	8	762	0.46	0.08	−0.83–0.98	0.41
Outliers removed^b^	—	—	—	—	—	—
Child molestation						
Original	20	1317	0.06	−0.08	−0.21–0.05	0.25
With study number as random effect	20	1317	0.07	−0.07	−0.20–0.06	0.25
Trim‐and‐fill	20	1317	0.06	−0.08	−0.21–0.05	0.25
Selection models	20	1317	0.23	−0.09	−0.54–0.37	0.27
Outliers removed^b^	—	—	—	—	—	—
Exhibitionism						
Original	3	317	0.06	0.15**	0.05–0.26	0.00
With study number as random effect	3	317	0.06	0.15**	0.05–0.26	0.00
Trim‐and‐fill	3	317	0.06	0.15**	0.05–0.26	0.00
Selection models^b^	—	—	—	—	—	—
Outliers removed^b^	—	—	—	—	—	—
Undifferentiated sexual aggression						
Original	107	38,999	0.03	0.37***	0.32–0.42	0.25
With study number as random effect	107	38,999	0.03	0.34***	0.27–0.40	0.25
Trim‐and‐fill	107	38,999	0.03	0.37***	0.32–0.42	0.25
Selection models	107	38,999	0.03	0.41***	0.35–0.48	0.22
Outliers removed	102	38,208	0.02	0.34***	0.30–0.39	0.22
Voyeurism						
Original	4	1314	0.07	0.38***	0.25–0.52	0.11
With study number as random effect	4	1314	0.10	0.35***	0.16–0.54	0.13
Trim‐and‐fill	4	1314	0.07	0.38***	0.25–0.52	0.11
Selection models^b^	—	—	—	—	—	—
Outliers removed^b^	—	—	—	—	—	—
Mixed sexual aggression						
Original	4	107	0.09	0.20*	0.02–0.39	0.00
With study number as random effect	4	107	0.09	0.20*	0.02–0.39	0.00
Trim‐and‐fill	4	107	0.09	0.20*	0.02–0.39	0.00
Selection models^b^	—	—	—	—	—	—
Outliers removed^b^	—	—	—	—	—	—
Sexual homicide						
Original	3	262	0.06	0.28***	0.17–0.39	0
With study number as random effect	3	262	0.06	0.28***	0.17–0.39	0
Trim‐and‐fill	3	262	0.06	0.28***	0.17–0.39	0
Selection models^b^	—	—	—	—	—	—
Outliers removed^b^	—	—	—	—	—	—
Sexual cyberbullying/harassment						
Original	3	1470	0.13	0.51***	0.25–0.77	0.23
With study number as random effect	3	1470	0.13	0.51***	0.25–0.77	0.23
Trim‐and‐fill	3	1470	0.13	0.51***	0.25–0.77	0.23
Selection models^b^	—	—	—	—	—	—
Outliers removed^b^	—	—	—	—	—	—
Sexual sadism						
Original	15	3074	0.06	0.33***	0.21–0.44	0.21
With study number as random effect	15	3074	0.07	0.34***	0.20–0.47	0.21
Trim‐and‐fill	15	3074	0.06	0.33***	0.21–0.44	0.21
Selection models	15	3074	0.10	0.31***	0.10–0.51	0.21
Outliers removed	14	3047	0.04	0.29***	0.21–0.37	0.12

Abbreviation: *k* = number of effect sizes.

^a^ When specifying study number as a random effect, the variance of random intercepts was denoted as sigma instead of tau.

^b^ Some rows are left blank because *k* was insufficient to run those models or there were no outliers.

* *p* < 0.05.

** *p* < 0.01.

*** *p* < 0.001.

For the overall relation between global psychopathy and overall SA, there was significant heterogeneity (general aggression: *Q*(166) = 11,044.18, *p <* 0.0001). Results also indicated that the magnitude of heterogeneity of effect sizes for the relation between global psychopathy and overall SA was large (*I* ^2^ = 98.09%), which supported the potential effect of moderators in explaining heterogeneity in the effect sizes. Different forms of SA as rendered by outcomes studied in the samples included in this meta‐analysis (i.e., rape, child molestation, undifferentiated SA, mixed SA (against both children and adults), sexual homicide, sexual cyberbullying/harassment, and sexual sadism) also showed significant heterogeneity (rape: *Q*(7) = 72.25, *p <* 0.0001, child molestation: *Q*(19) = 99.04, *p <* 0.0001, voyeurism: *Q*(3) = 11.67, *p* < 0.01, undifferentiated SA: *Q*(106) = 9100.94, *p <* 0.0001, sexual cyberbullying/harassment: *Q*(2) = 47.27, *p <* 0.0001, sexual sadism (*Q*(14) = 206.72, *p <* 0.0001), with the exception of exhibitionism: *Q*(2) = 3.72, *p >* 0.05, sexual homicide: *Q*(2) = 0.27, *p >* 0.05, and mixed SA *Q*(3) = 2.26, *p >* 0.05). Like with overall SA, the relations between global psychopathy and most of these different forms of SA evinced heterogeneity in effect sizes that was large in magnitude (rape: *I* ^2^ = 88.65%, child molestation: *I* ^2^ = 81.73%, voyeurism: *I* ^2^ = 80.17%, undifferentiated SA: *I* ^2^ = 98.31%, sexual cyberbullying/harassment: *I* ^2^ = 97.93%, sexual sadism: *I* ^2^ = 92.28%), supporting the possible effects of moderators in accounting for this heterogeneity, with the exception of exhibitionism (*I* ^2^ = 0.03%), sexual homicide (*I* ^2^ = 0.00%), and mixed SA (*I* ^2^ = 0.00%). Given that child molestation evinced such a different effect size from other forms of SA, the effect size was generated for psychopathy and overall SA *without* child molestation included. This increased the significant, positive association between psychopathy and general SA (*r* = 0.35); heterogeneity was slightly lower than when child molestation was included (*I* ^2^ = 97.81%).

We used funnel plots, the trim‐and‐fill method, and selection models to assess potential publication biases. As presented in Figure S2, funnel plots depicting effect sizes demonstrated symmetrical patterns for all 11 global psychopathy effects. Results from trim‐and‐fill analysis and step function selection models (Hedges 1992; Vevea and Woods 2005), which adjust for publication bias, yielded no significant differences between the original mean effect sizes and the mean effect sizes following the adjustments. Table 1 also displays effects when study number was specified in the model as a random effect as well as when outlier effects were removed from each meta‐analysis. Both methods rendered similar results to the original mean effect sizes.

### 2.2 Triarchic Psychopathy Effects

Table 2 and Table S3 depict findings from the moderator analysis at the triarchic level using rescaled relative weights (i.e., percentages of boldness, meanness, and disinhibition represented in a psychopathy measure) and raw relative weights, respectively. To account for between‐study differences in test dependence, study number was included as a random effect. A significant moderating effect was found for all triarchic traits. The weight of boldness in a psychopathy measure (i.e., the extent to which boldness is represented in a psychopathy measure) significantly moderated the effect of psychopathy on overall SA: the more boldness is captured in a psychopathy measure, the weaker the relation between psychopathy and overall SA. The weights of meanness and disinhibition in a psychopathy measure significantly, positively moderated the effect of psychopathy on SA, such that the more meanness or disinhibition is captured in a psychopathy measure, the stronger the relation between psychopathy and overall SA. These moderating effects, except for meanness, held when child molestation studies were excluded. When examining the raw relative weights, a significant, negative effect of boldness was found for general SA, including when child molestation studies were excluded: greater representation of boldness in a psychopathy measure weakens the psychopathy‐SA relationship. No significant moderating effects of triarchic psychopathy traits, using rescaled or raw relative weights, were found for child molestation or undifferentiated SA.

**TABLE 2:** Meta‐analytically derived relations between sexual aggression and psychopathy at the triarchic level: rescaled relative weights (i.e., percentages).

	*k*	*N*	SE	Weighted ES	*σ*
General sexual aggression					
Boldness	161	45,145	0.0009	−0.0030***	0.28
0%	—	—	—	0.35	—
25%	—	—	—	0.28	—
50%	—	—	—	0.20	—
75%	—	—	—	0.13	—
100%	—	—	—	0.05	—
Meanness	161	45,145	0.0022	0.0050*	0.28
0%				0.06	—
25%	—	—	—	0.19	—
50%	—	—	—	0.31	—
75%	—	—	—	0.44	—
100%	—	—	—	0.56	—
Disinhibition	161	45,145	0.0011	0.0032**	0.28
0%	—	—	—	0.16	—
25%	—	—	—	0.24	—
50%	—	—	—	0.32	—
75%	—	—	—	0.40	—
100%	—	—	—	0.48	—
General SA without CM					
Boldness	141	43,828	0.0009	−0.0029**	0.25
0%	—	—	—	0.39	—
25%	—	—	—	0.32	—
50%	—	—	—	0.25	—
75%	—	—	—	0.17	—
100%	—	—	—	0.10	—
Meanness	141	43,828	0.0022	0.0042	0.25
0%	—	—	—	0.14	—
25%	—	—	—	0.25	—
50%	—	—	—	0.35	—
75%	—	—	—	0.46	—
100%	—	—	—	0.56	—
Disinhibition	141	43,828	0.0011	0.0033**	0.25
0%	—	—	—	0.21	—
25%	—	—	—	0.29	—
50%	—	—	—	0.38	—
75%	—	—	—	0.46	—
100%	—	—	—	0.54	—
Child molestation					
Boldness	20	1317	0.0058	0.0078	0.24
0%	—	—	—	−0.30	—
25%	—	—	—	−0.11	—
50%	—	—	—	0.09	—
75%	—	—	—	0.29	—
100%	—	—	—	0.48	—
Meanness	20	1317	0.0085	−0.0046	0.26
0%	—	—	—	0.11	—
25%	—	—	—	−0.01	—
50%	—	—	—	−12	—
75%	—	—	—	−0.24	—
100%	—	—	—	−0.35	—
Disinhibition	20	1317	0.0106	−0.0207	0.22
0%	—	—	—	0.61	—
25%	—	—	—	0.09	—
50%	—	—	—	−0.43	—
75%	—	—	—	−0.94	—
100%	—	—	—	−1.46	—
Undifferentiated					
Boldness	104	37,755	0.0020	−0.0022	0.26
0%	—	—	—	0.39	—
25%	—	—	—	0.34	—
50%	—	—	—	0.28	—
75%	—	—	—	0.23	—
100%	—	—	—	0.17	—
Meanness	104	37,755	0.0024	0.0024	0.26
0%	—	—	—	0.24	—
25%	—	—	—	0.30	—
50%	—	—	—	0.36	—
75%	—	—	—	0.42	—
100%	—	—	—	0.48	—
Disinhibition	104	37,755	0.0030	0.0012	0.26
0%	—	—	—	0.30	—
25%	—	—	—	0.33	—
50%	—	—	—	0.36	—
75%	—	—	—	0.39	—
100%	—	—	—	0.42	—

Abbreviations: CM = child molestation; *k* = number of effect sizes; SA = sexual aggression.

* *p* < 0.05.

** *p* < 0.01.

*** *p* < 0.001.

### 2.3 Remaining Moderation Results

Moderation analyses examining several variables pertaining to demographics, measures used, and other study characteristics were also carried out. Table 3 displays findings for these remaining moderation results conducted for the meta‐analyses which have *k* ≥ 20 effect sizes. Table S4 displays remaining moderation results for the other meta‐analyses with fewer than 20 effect sizes (e.g., rape; sexual homicide). Of note, some moderation analyses could not be carried out for certain specific SA forms for reasons related to a lack of studies or levels of categorical moderators or too much missing data. Because a large number of moderating effects were investigated, the threshold for significance was adjusted to *α* < 0.01 for evaluating these results.

**TABLE 3:** Meta‐analytically derived relations between sexual aggression and psychopathy: remaining moderation results.

	*k*	*N*	Reference group	SE	Effect	*σ*
General sexual aggression						
How effect size produced	166	47,619				0.26
Compared groups			Direct corr	0.06	−0.20***	
Gender	161	47,056		0.00	0.00	0.28
Race	109	33,794		0.00	−0.00	0.23
Age	149	41,935		0.00	−0.00	0.27
Sample type	166	47,619				0.24
Student			Sex offenders	0.08	−0.17	
Community			Sex offenders	0.08	−0.15	
Forensic non‐SOs			Sex offenders	0.08	−0.39***	
Forensic and non‐forensic combin.			Sex offenders	0.10	−0.34***	
Country^a^	166	47,619				0.26
Singapore			United States	0.26	0.64	
Netherlands			United States	0.21	−0.69***	
SA measure source	166	47,619				0.28
Interview			Self‐report	0.37	−0.36	
Record review			Self‐report	0.05	−0.13**	
Combination			Self‐report	0.12	0.11	
SA measure self‐report vs. not			Self‐report	0.04	−0.12**	0.28
SA measure^a^	166	47,619				0.24
Clinician ratings/DSM criteria			Legal records	0.03	−0.16***	
Items measuring sexual cyberbullying/harassment			Legal records	0.17	0.50**	
MASORR			Legal records	0.07	0.36***	
MnSOST‐R			Legal records	0.04	0.37***	
RM2000/S			Legal records	0.05	0.20***	
SORAG			Legal records	0.04	0.60***	
Static			Legal records	0.04	0.32***	
SVR‐20			Legal records	0.04	0.69***	
VRAG‐R			Legal records	0.04	0.52***	
VRS‐SO			Legal records	0.12	0.56***	
SA risk assessment vs. not			Risk	0.03	−0.46***	0.25
SA measure: only SA items vs. not			Only SA items	0.02	0.07***	0.27
Psy measure source	166	47,619				0.27
Self‐report			Record review	0.08	−0.19	
Interview			Record review	0.17	−0.08	
Both interview and record review			Record review	0.09	−0.19	
Other combination			Record review	0.21	0.03	
Psy measure record review vs. not			Record review	0.08	−0.18	0.27
Psy measure^a^	166	47,619				0.28
PPI			PCL	0.08	−0.20**	
PCL vs. not			PCL	0.06	−0.08	0.27
Sex offender sample type	110	25,260				0.33
Rapists			Child SOs	0.05	0.19***	
Exhibitionists			Child SOs	0.08	0.17	
Undifferentiated SOs			Child SOs	0.05	0.59***	
Voyeurists			Child SOs	0.30	−0.03	
Mixed SOs			Child SOs	0.10	0.30**	
Sexual homicide offenders			Child SOs	0.17	0.63***	
SOs with sexual sadism			Child SOs	0.06	0.40***	
CSOs vs. not			Child SOs	0.04	0.37***	0.31
Victim class	166	47,619				0.26
Adults			Children	0.05	0.21***	
Mixed—adults and children			Children	0.10	0.30**	
Undifferentiated			Children	0.04	0.45***	
Children victims only vs. not	166	47,619	Children	0.03	0.34***	0.26
General sexual aggression without CM						
How effect size produced	146	46,302				0.24
Compared groups			Direct corr	0.06	−0.11	
Gender	143	45,822		0.00	0.00	0.25
Race	98	32,831		0.00	−0.00	0.22
Age	132	40,717		0.00	0.00	0.24
Sample type	146	46,340				0.21
Student			Sex offenders	0.07	−0.20**	
Community			Sex offenders	0.07	−0.18	
Forensic non‐SOs			Sex offenders	0.07	−0.37***	
Forensic and non‐forensic combin.			Sex offenders	0.11	−0.11	
Country	146	46,302				0.25
Singapore			United States	0.25	0.61	
SA measure source	146	46,302				0.25
Interview			Self‐report	0.35	−0.38	
Record review			Self‐report	0.04	−0.08	
Clinician ratings			Self‐report	0.27	−0.06	
Combination			Self‐report	0.11	0.10	
SA measure self‐report vs. not			Self‐report	0.04	−0.07	0.25
SA measure^a^	146	46,302				0.21
Clinician ratings/DSM criteria			Legal records	0.04	−0.11**	
Items measuring sexual cyberbullying/harassment			Legal records	0.15	0.43**	
MASORR			Legal records	0.07	0.31***	
MnSOST‐R			Legal records	0.04	0.33***	
RM2000/S			Legal records	0.05	0.15**	
SORAG			Legal records	0.04	0.56***	
Static			Legal records	0.04	0.28***	
SVR‐20			Legal records	0.04	0.64***	
VRAG‐R			Legal records	0.04	0.47***	
VRS‐SO			Legal records	0.12	0.50***	
SA risk assessment vs. not			Risk	0.03	−0.39***	0.22
SA measure: only SA items vs. not			Only SA items	0.02	0.08***	0.24
Psy measure source	146	46,302				0.24
Self‐report			Record review	0.08	−0.17	—
Interview			Record review	0.17	−0.01	—
Both interview and record review			Record review	0.08	−0.17	—
Other combination			Record review	0.19	−0.01	
Psy measure record review vs. not			Record review	0.07	−0.16	0.24
Psy measure^a^	146	46,302				0.24
PPI			PCL	0.07	−0.19**	
PCL vs. not			PCL	0.06	−0.08	0.24
Sex offender sample type	91	24,031				0.30
Rapists			Child SOs	0.11	−0.19	
Exhibitionists			Child SOs	0.16	−0.50**	
Undifferentiated SOs			Child SOs	0.15	−0.08	
Voyeurists			Child SOs	0.33	−0.69	
Mixed SOs			Child SOs	0.15	−0.14	
Sexual homicide offenders			Child SOs	0.20	0.03	
SOs with sexual sadism			Child SOs	0.15	−0.27	
CSOs vs. not			Child SOs	0.11	−0.17	0.29
Victim class	146	46,302				0.24
Adults			Children	0.11	−0.21	
Mixed—adults and children			Children	0.15	−0.16	
Undifferentiated			Children	0.12	−0.10	
Children victims only vs. not			Children	0.10	−0.18	0.24
Child molestation						
How effect size produced	20	1317				0.19
Compared groups			Direct corr	0.12	−0.13	
Gender^b^	—	—	—	—	—	—
Race	11	963		0.00	0.00	0.17
Age	17	1218		0.01	−0.01	0.35
Sample type	20	1317				0.19
Student			Sex offenders	0.24	0.16	—
Forensic non‐SOs			Sex offenders	0.13	−0.02	—
Forensic and non‐forensic combin.			Sex offenders	0.15	−0.16	—
Country	20	1317				0.18
Canada			United States	0.13	0.00	
Germany			United States	0.33	−0.08	
Italy			United States	0.22	0.16	
Netherlands			United States	0.18	−0.38	
South Korea			United States	0.28	0.13	
Switzerland			United States	0.24	0.02	
SA measure source	20	1317				0.20
Record review			Self‐report	0.18	−0.14	
Combination			Self‐report	0.27	−0.21	
SA measure	20	1317				0.20
Clinician ratings/DSM criteria			Legal records	0.17	0.16	
MASA			Legal records	0.24	0.24	
MSI			Legal records	0.25	0.09	
SA risk assessment vs. not^b^			—	—	—	—
SA measure: only SA items vs. not			Only SA items	0.12	0.16	0.18
Psy measure source	20	1317				0.29
Self‐report			Record review	0.24	−0.04	—
Interview			Record review	0.30	0.16	—
Both interview and record review			Record review	0.23	0.09	—
Psy measure record review vs. not			Record review	0.22	0.05	0.19
Psy measure	20	1317				0.18
LSRP			PCL	0.21	−0.43	—
PPI			PCL	0.15	−0.02	—
SRP			PCL	0.17	−0.08	—
PCL vs. not			PCL	0.12	−0.12	0.19
Sex offender sample type^b^	—	—	—	—	—	—
Victim class^b^	—	—	—	—	—	—
Undifferentiated						
How effect size produced	107	38,999				0.20
Compared groups			Direct corr	0.06	−0.08	
Gender	106	38,911		0.00	0.00	0.19
Race	70	27,563		0.00	0.00	0.17
Age	95	33,830		0.00	0.00	0.19
Sample type	107	38,999				0.14
Student			Sex offenders	0.05	−0.27***	
Community			Sex offenders	0.05	−0.24***	
Forensic non‐SOs			Sex offenders	0.06	−0.44***	
Forensic and non‐forensic combin.			Sex offenders	0.08	−0.20*	
Country^a^	107	38,999				0.20
Austria			United States	0.20	0.49	
SA measure source	107	38,999				0.19
Interview			Self‐report	0.31	−0.25	
Record review			Self‐report	0.05	0.09	
Combination			Self‐report	0.09	0.17	
SA measure self‐report vs. not			Self‐report	0.05	0.10	0.19
SA measure^a^	107	38,999				0.15
ISOS‐PR			Legal records	0.16	0.32	
MASORR			Legal records	0.08	0.24**	
MnSOST‐R			Legal records	0.04	0.25**	
SORAG			Legal records	0.05	0.50***	
Static			Legal records	0.04	0.20***	
SVR‐20			Legal records	0.05	0.50***	
VRAG‐R			Legal records	0.05	0.42***	
VRS‐SO			Legal records	0.12	0.38**	
SA risk assessment vs. not			Risk	0.04	−0.26***	0.16
SA measure: only SA items vs. not			Only SA items	0.03	0.10***	0.18
Psy measure source	107	38,999				0.18
Self‐report			Record review	0.07	−0.21**	
Interview			Record review	0.17	0.00	
Both interview and record review			Record review	0.08	−0.11	
Other combination			Record review	0.20	−0.01	
Psy measure record review vs. not			Record review	0.07	−0.17	0.19
Psy measure	107	38,999				0.19
Dirty Dozen			PCL	0.15	−0.19	
LSRP			PCL	0.07	−0.13	
PPI			PCL	0.08	−0.17	
SD3‐P			PCL	0.12	−0.25	
SRP			PCL	0.07	−0.12	
TriPM			PCL	0.09	−0.09	
YPI‐S			PCL	0.19	−0.08	
PCL vs. not			PCL	0.05	−0.15**	0.18
Sex offender sample type	63	20,907				0.24
Rapists			Undiff	0.22	0.12	
Child Molesters			Undiff	0.18	0.23	
Sexual homicide offenders			Undiff	0.25	0.39	
CSOs vs. not			Child SOs	0.13	−0.15	0.24
Victim class	107	38,999				0.20
Adults			Undiff	0.11	−0.02	
Children			Undiff	0.13	0.17	
Children victims only vs. not	107	38,999	Children	0.12	−0.17	0.19

*Note:* ***p < 0.001, **p < 0.01; *p* < 0.05 is not signified because a *p* < 0.01 significance level was used for these moderation analyses.

Abbreviations: Corr = correlation; CSOs = child sexual offenders; DSM = Diagnostic and Statistical Manual of Mental Disorders; ISOS‐PR = Interpersonal Sexual Objectification Scale‐Perpetration Revised; *k* = number of effect sizes; LSRP = Levenson Self‐Report Psychopathy Scale; MASA = Multidimensional Assessment of Sexual Aggression; MASORR = Multifactorial Assessment of Sex Offender Risk for Recidivism; MnSOST‐*R* = Minnesota Sex Offender Screening Tool‐Revised; MSI = Multiphasic Sexual Inventory; PCL = Psychopathy Checklist; PPI = Psychopathic Personality Inventory; RM2000/S = Risk Matrix 2000/Sexual Offending; SA = sexual aggression; SD3‐*P* = Short Dark Triad‐Psychopathy Scale; SORAG = Sex Offender Risk Appraisal Guide; SOs = sexual offenders; SRP = Self‐Report Psychopathy Scale; SVR‐20 = Sexual Violence Risk‐20; TriPM = Triarchic Psychopathy Measure; Undiff = undifferentiated sexual offenders; VRAG‐*R* = Violence Risk Appraisal Guide‐Revised; VRS‐SO = Violence Risk Scale: Sexual Offender Version; YPI‐S = Youth Psychopathic Traits Inventory.

^a^ Due to numerous levels of this categorical moderator and space constraints, only results for levels that were significantly different from the reference group are included here. When no significant differences were found, only effects that approached significance are listed.

^b^ This analysis could not be conducted due to too few studies, too much missing data for the moderator variable, and/or lack of variability (e.g., for every child molestation study, gender was 100% male).

A few follow‐up exploratory analyses, based on a few significant moderating effects, were conducted to see if these effects differed when (a) including only effect sizes produced when the source of the psychopathy measure was record review and the psychopathy measure was the PCL, separately; (b) including only effect sizes produced when the source of the SA measure was self‐report, a risk assessment, and contained non‐SA items (e.g., an item indicating a history of non‐sexual institutional misconduct), separately; (c) including only effect sizes for child sex offender samples and child victims, separately; and (d) comparing when effect sizes were rendered from direct correlations versus comparing groups on mean scores.

#### 2.3.1 Age, Gender, Race, and Country

There were no significant moderating effects of age, gender, or race on the relation between psychopathy and SA. The only moderating effect of country meeting our *α* < 0.01 threshold was found for the Netherlands (*B* = −0.69, *p* < 0.001); compared to studies conducted with samples in the United States, studies conducted with samples in the Netherlands significantly weaken the relation between psychopathy and general SA.

#### 2.3.2 Overall Sample Type

Sample type, with sexual offenders as the reference group, significantly moderated the relationship between global psychopathy and general SA, including when child molestation was excluded, as well as between psychopathy and undifferentiated aggression. Specifically, compared to sexual offenders, non‐sexual offenders and samples comprising a combination of forensic and non‐forensic participants significantly weakened psychopathy's effect on general SA (*B* = −0.38, *p* < 0.001; *B* = −0.34, *p* < 0.001, respectively). When child molestation was excluded, compared to sexual offenders, students and non‐sexual offenders significantly weakened psychopathy's effect on general SA (*B* = −0.20, *p* < 0.01; *B* = −0.37, *p* < 0.001, respectively). Additionally, compared to sexual offenders, students, community participants, and non‐sexual offenders significantly weakened psychopathy's effect on undifferentiated SA (*B* = −0.27, *p* < 0.001; *B* = −0.24, *p* < 0.001; *B* = −0.44, *p* < 0.001, respectively). Thus, sexual offenders as sample type strengthened these effect sizes compared to the other sample types. No such effects were found for child molestation.

#### 2.3.3 Sexual Aggression Measure Source and Specific Measures

Compared to self‐report SA measures, record review measures, and all non‐self‐report measures together, significantly weakened the relationship between global psychopathy and general SA (*B* = −0.13, *p* < 0.01; *B* = −0.12, *p* < 0.01, respectively). All remaining moderating effects pertaining to SA measure source were not significant. Several specific SA measures positively, significantly moderated psychopathy's effect on general SA, such as items measuring sexual cyberbullying/harassment (*B* = 0.50, *p* < 0.01) and risk assessments, such as VRAG‐R (*B* = 0.52, *p* < 0.001) and VRS‐SO (*B* = 0.56, *p* < 0.001; see Table 3). The effects were seen when child molestation studies were excluded and in undifferentiated SA. Conversely, clinician ratings/DSM criteria as the measure were a significant, negative moderator for general SA (*B* = −0.16, *p* < 0.001), including without child molestation studies. No significant moderation effects of SA measure were found for child molestation.

Compared to non‐risk‐assessment SA measures, risk assessment SA measures significantly strengthened psychopathy's association with general SA (*B* = 0.46, *p* < 0.001), including when child molestation was excluded (*B* = 0.33, *p* < 0.001), as well as with undifferentiated SA (*B* = 0.39, *p* < 0.001). Additionally, for psychopathy's effect on general SA, general SA without child molestation included, and undifferentiated SA, SA measures containing additional items not directly measuring SA had a significantly stronger positive effect compared to measures with only items pertaining to SA (*B* = 0.09, *p* < 0.001; *B* = 0.08, *p* < 0.001; *B* = 0.10, *p* < 0.001, respectively). Such moderating effects were not found for child molestation.

#### 2.3.4 Psychopathy Measure Source and Specific Measures

For undifferentiated SA, compared to self‐report psychopathy measures, record review psychopathy measures significantly strengthened the association (*B* = 0.21, *p* < 0.01). No other significant moderating effects were found for psychopathy measure source. For general SA, including without child molestation studies, compared to PPI, PCL‐R significantly strengthened the psychopathy‐SA association (*B* = 0.20 and 0.19, respectively, *p* < 0.01). When PCL‐R was compared to all other psychopathy measures, it significantly strengthened the association between psychopathy and undifferentiated SA (*B* = 0.15, *p* < 0.01). No other significant moderating effects were found for psychopathy measure.

#### 2.3.5 Sexual Offender Sample Type

Sexual offender sample type (i.e., rapists, exhibitionists, child sexual offenders, undifferentiated sexual offenders, voyeurists, mixed sexual offenders, sexual homicide offenders, and sexually sadistic sexual offenders) was examined as a potential moderator of psychopathy's effect on general SA, general SA without child molestation, child molestation, and undifferentiated SA. For studies that used sexual offender samples, compared to child sexual offenders as a reference group, rapists (*B* = 0.19, *p* < 0.001), undifferentiated sexual offenders (*B* = 0.59, *p* < 0.001), mixed sexual offenders (*B* = 0.30, *p* < 0.01), sexual homicide offenders (*B* = 0.63, *p* < 0.001), and sexually sadistic sexual offenders (*B* = 0.40, *p* < 0.001) evinced significant, positive moderating effects on psychopathy and general SA. When comparing child sexual offenders to all other sexual offender sample types together, non‐child sexual offender samples significantly strengthened the association between psychopathy and general SA (*B* = 0.37, *p* < 0.001). For general SA without studies examining child molestation as the outcome, compared to child sexual offenders who completed undifferentiated SA outcome measures, using a sexual offender sample of exhibitionists significantly weakened the relation (*B* = −0.50, *p* < 0.01). No significant effect of sexual offender sample type was found for undifferentiated SA.

#### 2.3.6 Victim Class

SA victim classes of adults only, children only, mixed (children and adults), and undifferentiated were examined as potential moderators of psychopathy's effect on SA. With children as a reference group, all other victim classes evinced significant, positive moderating effects for psychopathy and general SA (Adults: *B* = 0.21, *p* < 0.001; Mixed: *B* = 0.30, *p* < 0.01; Undifferentiated: *B =* 0.45, *p* < 0.001). Correspondingly, when pitting child victims against all other victim classes, there was also a significant, positive moderating effect of non‐child victim class on psychopathy and general SA (*B* = 0.34, *p* < 0.001). No other significant moderating effects of victim class were found.

#### 2.3.7 How Effect Size Was Produced

The method used to obtain an effect size was examined—namely, whether the effect came from a direct correlation between two measures or if it was derived by comparing two groups on mean scores and standard deviations of a given measure. The latter situation most often occurred when a subsample of sexual offenders was compared to a control group, usually non‐sexual offenders, on mean scores on the PCL‐R. Compared to direct correlations, comparing groups significantly weakened the association between psychopathy and general SA (*B* = −0.20, *p* < 0.001), but not other SA forms with *k* ≥ 20 effect sizes.

## 3 Discussion

Results from the current meta‐analysis demonstrate that global psychopathy scores evince a positive, moderate, significant association with SA. This effect was also seen in more specific forms of SA, including undifferentiated SA, exhibitionism, voyeurism, mixed SA, sexual homicide, sexual cyberbullying/harassment, and sexual sadism. In other words, all forms of SA showed a significant association with psychopathy except for rape and child molestation. Our effect sizes were generally of a similar magnitude to psychopathy's relations with other relevant outcomes, including instrumental violence (*r* = 0.36; Blais et al. 2014), reactive violence (*r* = 0.35; Blais et al. 2014), and laboratory‐based aggression (*r* = 0.23; Hyatt et al. 2019).

Unlike other forms of SA, child molestation evinced almost no relation with psychopathy, and when child molestation was parsed out from general SA, the effect size increased from 0.30 to 0.35. This agrees with previous research which found that child molestation was negatively related to antagonism, the other pole of Agreeableness from the FFM and analogous to meanness in the triarchic model of psychopathy, in contrast to other forms of antisocial behavior (Vize et al. 2019). While *I* ^2^ indicated significant heterogeneity among child molestation studies, no moderation effects that we investigated were significant. Future meta‐analyses could investigate more nuanced factors influencing psychopathy in child molestation, such as perpetrator phenotypes—affiliative (e.g., relatives) vs. non‐affiliative (e.g., strangers), which tend to differ in approach (e.g., grooming vs. coercion; e.g., Marshall et al. 2015), offense type (e.g., contact offenses vs. viewing child sexual exploitation material; Lim et al. 2021), and victim age (e.g., pubescent vs. prepubescent; e.g., Kalichman 1991).

The effect sizes for psychopathy and rape were positive but smaller than expected. Additionally, while the effect size for psychopathy and sexual homicide was moderate and statistically significant, it was much smaller than that reported in a recent meta‐analysis by Fox and DeLisi (2019; *r* = 0.71). This discrepancy is not entirely surprising, as Fox and DeLisi (2019) used substantially different methods, leading to different studies and effect sizes being analyzed. Specifically, they included studies without a comparison group, using a non‐offender community PCL‐R norm score to calculate effect sizes, which will serve to maximize effect sizes. They also only used studies that assessed psychopathy with PCL measures. Given our finding that PCL measures significantly strengthened the psychopathy–undifferentiated SA association, and considering that non‐offender community samples generally have a restricted range of PCL scores (e.g., Babiak et al. 2010; Hare 2003), their larger effect size is to be expected. In contrast, we used the comparison groups provided within studies that met our eligibility criteria, and when studies did not have a comparison group, we excluded those studies rather than use a norm. We recommend that future meta‐analyses utilizing norm‐based effect size calculations use a comparison group more aligned with the target group under study. For example, sexual offenders may best be compared with non‐sexual offenders rather than community members.

### 3.1 Moderation Results

At the triarchic level of psychopathy, findings indicated that psychopathy measures with greater representation of meanness and disinhibition relate more strongly to SA overall. The effects of meanness and disinhibition in this moderation analysis suggest that these traits are more important for predicting SA overall and are consistent with findings from meta‐analyses examining aggression in general (Jones et al. 2011; Vize et al. 2019) and intimate partner violence (Collison and Lynam 2021) which identify Agreeableness and Conscientiousness (the FFM counterparts of Meanness and Disinhibition) as the strongest correlates. These results are also consistent with previous findings that SA is linked to the diminished ability to inhibit sexual drive in response to sexually coercive situations (Knight and Guay 2006) and a lack of responsivity to others' distress cues (Blair et al. 2005), reflecting a lack of self‐control and empathy (or low cognitive‐affective control and affiliative capacity, per the triarchic model of psychopathy; e.g., Patrick and Drislane 2015), that is relevant to disinhibition and meanness, respectively. Surprisingly, the meanness effect with general sexual aggression was no longer statistically significant when child molestation studies were excluded. We also found that greater representation of boldness in a psychopathy measure significantly weakened the association between psychopathy and general SA, including when child molestation was excluded. This result aligns with findings that boldness is more strongly associated with adaptive outcomes than maladaptive outcomes that have led to questions regarding its relevance to the psychopathy construct (e.g., Miller et al. 2020; Sleep et al. 2019; for an alternative viewpoint, see Lilienfeld et al. 2016; Patrick 2022).

Several sample and measurement characteristics emerged as significant moderators across multiple forms of SA. The largest of these effects was found for SA risk assessments, sexual offender sample type, and victim class. Specifically, risk assessment SA measures significantly strengthened the relation between psychopathy and SA. Risk assessments for sexual recidivism often include items for sexual offending, other antisocial behavior, and sometimes psychopathy or callous traits, all of which correlate with psychopathy and may inflate effect sizes via some degree of criterion contamination. Additionally, compared to child sexual offenders and child victims, almost all other types of sexual offenders and victim classes significantly strengthened the relation between psychopathy and SA. These results highlight that child molestation is different from other forms of SA in its relation to psychopathy.

## 4 Limitations

The current meta‐analysis has multiple limitations. First, there was a high degree of variability in the characteristics of studies used for the meta‐analysis, including those pertaining to methods of measurement and sample demographics. While this variability was addressed by examining many moderating variables in the analyses, methodological and sample characteristic differences may have greatly contributed to significant heterogeneity across studies. It was also less than optimal that a minimal number of studies reported effect sizes for certain forms of SA, namely, mixed SA, sexual homicide, and sexual cyberbullying/harassment, and that some moderation analyses could not be run for all SA forms.

Additionally, while we worked to avoid duplicate/overlapping samples in the present meta‐analysis, there is room for inaccuracy due, in part, to how common it is for researchers to reuse whole or parts of datasets in ways that are not always transparently reported. The study also has limitations pertaining to the triarchic psychopathy moderation analysis. Namely, some of the relative weights that were used for the triarchic psychopathy traits moderator analysis were generated from samples that were smaller than ideal. Specifically, those for PPI‐SF were derived from a sample of *N* = 141, PCL‐R from a sample of *N* = 169, and PCL:SV from a sample of *N* = 92.

Additionally, the triarchic psychopathy moderator analysis was restricted by whatever amount of information extant psychopathy measures have to offer at the triarchic level. For instance, assessing the effect of 100% meanness represented in a psychopathy measure on SA is not possible because no extant psychopathy measure comprises that level of meanness nor likely ever will considering the high overlap of personality dimensions. Furthermore, the relative weights analysis cannot speak to the direct relationship between SA and the triarchic psychopathy traits themselves (e.g., we cannot conclude that boldness is negatively associated with SA). Rather, it indicates the effect of each trait on the relation between SA and psychopathy via the extent to which the trait is represented in a psychopathy measure (e.g., greater representation of boldness in a measure weakened the association between SA and psychopathy).

## 5 Conclusion

In conclusion, this meta‐analysis demonstrated that psychopathy relates significantly and moderately to multiple forms of SA, including general SA, undifferentiated SA, exhibitionism, voyeurism, mixed SA, sexual homicide, sexual cyberbullying/harassment, and sexual sadism. Results indicated significant heterogeneity among studies and several moderators that help explain nuances of psychopathy's effect on SA. The results from the current study suggest that examining psychopathy at a more granular level might be useful but that more studies examining triarchic psychopathy directly in relation to SA are needed. Understanding how such mechanisms relate to SA can inform prevention of and treatment for damaging sexual acts.

## Author Contributions

The authors confirm contributions to the paper as follows: Study conceptualization and design: M.P.W. and D.R.L.; data curation, formal analysis, interpretation of results, and draft manuscript preparation: M.P.W.; coding validation: K.V.T.; statistical consultation: T.V.D. and D.R.L.; manuscript editing: M.P.W., T.V.D., K.V.T., D.R.L., and J.D.M. All authors reviewed the results and approved the final version of the manuscript.

## Disclosure

Permission to Reproduce Material From Other Sources: No material from other sources were reproduced. Investigators of unpublished data provided the data and permission to include them in this meta‐analysis.

## Ethics Statement

This meta‐analysis did not require ethical approval, as it only conducted secondary analysis of previous published and unpublished studies in which informed consent was obtained by the original investigators.

## Conflicts of Interest

The authors declare no conflicts of interest.

## Supporting information


**Data S1:** jopy70017‐sup‐0001‐DataS1.docx.

## Data Availability

The data and code that support the findings of this study are available on OSF: https://osf.io/u6jze/?view_only=3ab83176bffc47a68f3ee6d7c10706ea.

## Endnotes

^1^
We preregistered that we would conduct these moderation analyses with all of the more specific forms of sexual aggression but found that some forms had too few effect sizes to run such analyses and that there were too many results to discuss within the text. We table the non‐triarchic psychopathy moderation results for sexual aggression forms with less than 20 effect sizes in the Supporting Information only.

^2^
We also planned to meta‐analyze the relation between SA and the triarchic domains but did not due to too few studies (i.e., two) reporting these relations.

^3^
We preregistered that we would also correct for artificial dichotomization, as we expected many studies would use the PCL‐R cut‐off score to diagnose psychopathy but ultimately did not because < 5 studies used this method.

## References

[jopy70017-bib-0001] Abbey, A. , A. J. Jacques‐Tiura , and J. M. LeBreton . 2011. “Risk Factors for Sexual Aggression in Young Men: An Expansion of the Confluence Model.” Aggressive Behavior 37, no. 5: 450–464. 10.1002/ab.20399. 21678429 PMC3139770

[jopy70017-bib-0002] Abbey, A. , M. R. Parkhill , R. BeShears , A. M. Clinton‐Sherrod , and T. Zawacki . 2006. “Cross‐Sectional Predictors of Sexual Assault Perpetration in a Community Sample of Single African American and Caucasian Men.” Aggressive Behavior 32, no. 1: 54–67. 10.1002/ab.20107. 26435555 PMC4589184

[jopy70017-bib-0003] Abbey, A. , M. R. Parkhill , A. M. Clinton‐Sherrod , and T. Zawacki . 2007. “A Comparison of Men Who Committed Different Types of Sexual Assault in a Community Sample.” Journal of Interpersonal Violence 22, no. 12: 1567–1580. 10.1177/0886260507306489. 17993642 PMC4484268

[jopy70017-bib-0004] Abbey, A. , R. Wegner , J. Pierce , and A. J. Jacques‐Tiura . 2012. “Patterns of Sexual Aggression in a Community Sample of Young Men: Risk Factors Associated With Persistence, Desistance, and Initiation Over a 1‐Year Interval.” Psychology of Violence 2, no. 1: 1–15. 10.1037/a0026346. 22272382 PMC3262661

[jopy70017-bib-0005] American Psychiatric Association . 2013. Diagnostic and Statistical Manual of Mental Disorders: DSM‐5. 5th ed. American Psychiatric Association.

[jopy70017-bib-0006] Babiak, P. , C. S. Neumann , and R. D. Hare . 2010. “Corporate Psychopathy: Talking the Walk.” Behavioral Sciences & the Law 28, no. 2: 174–193. 10.1002/bsl.925. 20422644

[jopy70017-bib-0007] Barbaree, H. , C. Langton , and E. Peacock . 2006. “Sexual Offender Treatment for Psychopaths: Is It Harmful?” In Sexual Offender Treatment: Controversial Issues, edited by W. L. Marshall , Y. M. Fernandez , L. E. Marshall , and G. A. Serran . John Wiley & Sons, Ltd.

[jopy70017-bib-0008] Blair, J. , D. R. Mitchell , and K. Blair . 2005. The Psychopath: Emotion and the Brain. Blackwell Pub.

[jopy70017-bib-0009] Blais, J. , E. Solodukhin , and A. E. Forth . 2014. “A Meta‐Analysis Exploring the Relationship Between Psychopathy and Instrumental Versus Reactive Violence.” Criminal Justice and Behavior 41, no. 7: 797–821. 10.1177/0093854813519629.

[jopy70017-bib-0010] Bonta, J. , and D. A. Andrews . 2023. The Psychology of Criminal Conduct. 7th ed. Routledge. 10.4324/9781003292128.

[jopy70017-bib-0011] Brazil, K. J. , S. Roy , K. V. Bubeleva , and C. S. Neumann . 2023. “Sexual Assault Proclivity and Sexual Aggression in College Men: Associations With Psychopathic Traits and Sex Drive.” Journal of Criminal Justice 87: 102089. 10.1016/j.jcrimjus.2023.102089.

[jopy70017-bib-0012] Brown, A. R. , M. A. Dargis , A. C. Mattern , M. A. Tsonis , and J. P. Newman . 2015. “Elevated Psychopathy Scores Among Mixed Sexual Offenders: Replication and Extension.” Criminal Justice and Behavior 42, no. 10: 1032–1044. 10.1177/0093854815575389.

[jopy70017-bib-0013] Cleckley, H. 1941. The Mask of Sanity: An Attempt to Reinterpret the So‐Called Psychopathic Personality. Mosby.

[jopy70017-bib-0015] Collison, K. L. , and D. R. Lynam . 2021. “Personality Disorders as Predictors of Intimate Partner Violence: A Meta‐Analysis.” Clinical Psychology Review 88: 102047. 10.1016/j.cpr.2021.102047. 34130046

[jopy70017-bib-0016] Collison, K. L. , D. R. Lynam , and J. D. Miller . 2023. “The Triarchic Psychopathy Model Is Embedded Within the Five Factor Model: No Need for Reconfiguration.” Journal of Psychopathology and Behavioral Assessment 45, no. 4: 1034–1045. 10.1007/s10862-023-10080-6.

[jopy70017-bib-0501] Collison, K. L. , J. D. Miller , and D. R. Lynam . 2021. “Examining the Factor Structure and Validity of the Triarchic Model of Psychopathy Across Measures.” Personality Disorders: Theory, Research, and Treatment 12, no. 2: 115–126. 10.1037/per0000394. 32105104

[jopy70017-bib-0017] Crego, C. , and T. A. Widiger . 2015. “Psychopathy and the DSM.” Journal of Personality 83, no. 6: 665–677. 10.1111/jopy.12115. 25039353

[jopy70017-bib-0020] DeGue, S. , D. DiLillo , and M. Scalora . 2010. “Are All Perpetrators Alike? Comparing Risk Factors for Sexual Coercion and Aggression.” Sexual Abuse: A Journal of Research and Treatment 22, no. 4: 402–426. 10.1177/1079063210372140. 20693517

[jopy70017-bib-0021] Drislane, L. E. , S. J. Brislin , S. Jones , and C. J. Patrick . 2018. “Interfacing Five‐Factor Model and Triarchic Conceptualizations of Psychopathy.” Psychological Assessment 30, no. 6: 834–840. 10.1037/pas0000544. 29172554

[jopy70017-bib-0022] Du, T. V. , J. D. Miller , and D. R. Lynam . 2022. “The Relation Between Narcissism and Aggression: A Meta‐Analysis.” Journal of Personality 90, no. 4: 574–594. 10.1111/jopy.12684. 34689345

[jopy70017-bib-0023] Duval, S. , and R. Tweedie . 2000. “Trim and Fill: A Simple Funnel‐Plot‐Based Method of Testing and Adjusting for Publication Bias in Meta‐Analysis.” Biometrics 56, no. 2: 455–463. 10.1111/j.0006-341X.2000.00455.x. 10877304

[jopy70017-bib-0025] Fox, B. , and M. DeLisi . 2019. “Psychopathic Killers: A Meta‐Analytic Review of the Psychopathy‐Homicide Nexus.” Aggression and Violent Behavior 44: 67–79. 10.1016/j.avb.2018.11.005.

[jopy70017-bib-0027] Hall, J. R. , L. E. Drislane , C. J. Patrick , M. Morano , S. O. Lilienfeld , and N. G. Poythress . 2014. “Development and Validation of Triarchic Construct Scales From the Psychopathic Personality Inventory.” Psychological Assessment 26, no. 2: 447–461. 10.1037/a0035665. 24447280 PMC4147944

[jopy70017-bib-0028] Hare, R. D. 1980. “A Research Scale for the Assessment of Psychopathy in Criminal Populations.” Personality and Individual Differences 1, no. 2: 111–119.

[jopy70017-bib-0029] Hare, R. D. 1991. The Hare Psychopathy Checklist‐Revised: Manual. Multi‐Health Systems, Incorporated.

[jopy70017-bib-0030] Hare, R. D. 2003. The Hare Psychopathy Checklist‐Revised. 2nd ed. Multi‐Health Systems.

[jopy70017-bib-0031] Hare, R. D. , and C. S. Neumann . 2008. “Psychopathy as a Clinical and Empirical Construct.” Annual Review of Clinical Psychology 4, no. 1: 217–246. 10.1146/annurev.clinpsy.3.022806.091452. 18370617

[jopy70017-bib-0032] Hawes, S. W. , M. T. Boccaccini , and D. C. Murrie . 2013. “Psychopathy and the Combination of Psychopathy and Sexual Deviance as Predictors of Sexual Recidivism: Meta‐Analytic Findings Using the Psychopathy Checklist—Revised.” Psychological Assessment 25, no. 1: 233–243. 10.1037/a0030391. 23088204

[jopy70017-bib-0033] Hedges, L. V. 1992. “Modeling Publication Selection Effects in Meta‐Analysis.” Statistical Science 7, no. 2: 246–255. 10.1214/ss/1177011364.

[jopy70017-bib-0034] Higgins, J. P. T. 2003. “Measuring Inconsistency in Meta‐Analyses.” British Medical Journal 327, no. 7414: 557–560. 10.1136/bmj.327.7414.557. 12958120 PMC192859

[jopy70017-bib-0035] Hildebrand, M. , C. de Ruiter , and V. de Vogel . 2004. “Psychopathy and Sexual Deviance in Treated Rapists: Association With Sexual and Nonsexual Recidivism.” Sexual Abuse: A Journal of Research and Treatment 16, no. 1: 1–24. 10.1177/107906320401600101. 15017823

[jopy70017-bib-0036] Hyatt, C. S. , A. Zeichner , and J. D. Miller . 2019. “Laboratory Aggression and Personality Traits: A Meta‐Analytic Review.” Psychology of Violence 9, no. 6: 675–689. 10.1037/vio0000236.

[jopy70017-bib-0037] Jones, S. E. , J. D. Miller , and D. R. Lynam . 2011. “Personality, Antisocial Behavior, and Aggression: A Meta‐Analytic Review.” Journal of Criminal Justice 39, no. 4: 329–337. 10.1016/j.jcrimjus.2011.03.004.

[jopy70017-bib-0038] Kalichman, S. C. 1991. “Psychopathology and Personality Characteristics of Criminal Sexual Offenders as a Function of Victim Age.” Archives of Sexual Behavior 20, no. 2: 187–197. 10.1007/BF01541943. 2064542

[jopy70017-bib-0039] Knight, R. A. , and F. J. Graham . 2017. “Hypersexuality: Equifinal, Cohesive, Clinical Presentation or Symptom Cluster With Multiple Underlying Mechanisms?” Archives of Sexual Behavior 46, no. 8: 2261–2264. 10.1007/s10508-017-1089-z. 28986708

[jopy70017-bib-0040] Knight, R. A. , and J. Guay . 2006. “The Role of Psychopathy in Sexual Coercion Against Women.” In Handbook of Psychopathy, edited by C. J. Patrick , 512–532. Guilford.

[jopy70017-bib-0041] Knight, R. A. , and J.‐P. Guay . 2018. “The Role of Psychopathy in Sexual Coercion Against Women: An Update and Expansion.” In Handbook of Psychopathy, edited by C. J. Patrick , 662–681. Guilford Press.

[jopy70017-bib-0042] Krahé, B. 2020. The Social Psychology of Aggression. 3rd ed. Routledge. 10.4324/9780429466496.

[jopy70017-bib-0044] Levenson, M. R. , K. A. Kiehl , and C. M. Fitzpatrick . 1995. “Assessing Psychopathic Attributes in a Noninstitutionalized Population.” Journal of Personality and Social Psychology 68, no. 1: 151–158. 10.1037/0022-3514.68.1.151. 7861311

[jopy70017-bib-0045] Lilienfeld, S. O. , and B. P. Andrews . 1996. “Development and Preliminary Validation of a Self‐Report Measure of Psychopathic Personality Traits in Noncriminal Populations.” Journal of Personality Assessment 66, no. 3: 488–524. 10.1207/s15327752jpa6603_3. 8667144

[jopy70017-bib-0502] Lilienfeld, S. O. , S. F. Smith , K. C. Sauvigné , et al. 2016. “Is Boldness Relevant to Psychopathic Personality? Meta‐Analytic Relations with Non‐Psychopathy Checklist‐Based Measures of Psychopathy.” Psychological Assessment 28, no. 10: 1172–1185. 10.1037/pas0000244. 26619088

[jopy70017-bib-0046] Lilienfeld, S. O. , and M. R. Widows . 2005. Psychopathic Personality Inventory‐Revised. Psychological Assessment Resources, Inc. 10.1037/pas0000032 25436663

[jopy70017-bib-0047] Lim, Y. Y. , S. Wahab , J. Kumar , F. Ibrahim , and M. R. Kamaluddin . 2021. “Typologies and Psychological Profiles of Child Sexual Abusers: An Extensive Review.” Children (Basel) 8, no. 5: 333. 10.3390/children8050333. 33922985 PMC8146192

[jopy70017-bib-0048] Lipsey, M. W. , and D. B. Wilson . 2001. Practical Meta‐Analysis. Sage Publications.

[jopy70017-bib-0049] Lynam, D. R. , E. T. Gaughan , J. D. Miller , D. J. Miller , S. Mullins‐Sweatt , and T. A. Widiger . 2011. “Assessing the Basic Traits Associated With Psychopathy: Development and Validation of the Elemental Psychopathy Assessment.” Psychological Assessment 23, no. 1: 108–124. 10.1037/a0021146. 21171784

[jopy70017-bib-0051] Lynam, D. R. , and J. D. Miller . 2015. “Psychopathy From a Basic Trait Perspective: The Utility of a Five‐Factor Model Approach: FFM Psychopathy.” Journal of Personality 83, no. 6: 611–626. 10.1111/jopy.12132. 25204751

[jopy70017-bib-0052] Lynam, D. R. , E. D. Sherman , D. Samuel , J. D. Miller , L. R. Few , and T. A. Widiger . 2013. “Development of a Short Form of the Elemental Psychopathy Assessment.” Assessment 20, no. 6: 659–669. 10.1177/1073191113502072. 23996849

[jopy70017-bib-0054] Marshall, W. L. 1992. “The Social Value of Treatment for Sexual Offenders.” Canadian Journal of Human Sexuality 1: 109–114.

[jopy70017-bib-0055] Marshall, W. L. , S. Smallbone , and L. E. Marshall . 2015. “A Critique of Current Child Molester Subcategories: A Proposal for an Alternative Approach.” Psychology, Crime & Law 21, no. 3: 205–218. 10.1080/1068316X.2014.925724.

[jopy70017-bib-0057] Miller, J. D. , and D. R. Lynam . 2012. “An Examination of the Psychopathic Personality Inventory's Nomological Network: A Meta‐Analytic Review.” Personality Disorders, Theory, Research, and Treatment 3, no. 3: 305–326. 10.1037/a0024567. 22452764

[jopy70017-bib-0058] Miller, J. D. , C. E. Sleep , M. L. Crowe , and D. R. Lynam . 2020. “Psychopathic Boldness: Narcissism, Self‐Esteem, or Something in Between?” Personality and Individual Differences 155: 109761. 10.1016/j.paid.2019.109761.

[jopy70017-bib-0059] Moher, D. , A. Liberati , J. Tetzlaff , and D. G. Altman . 2009. “Preferred Reporting Items for Systematic Reviews and Meta‐Analyses: The PRISMA Statement.” Journal of Clinical Epidemiology 62, no. 10: 1006–1012. 10.1016/j.jclinepi.2009.06.005. 19631508

[jopy70017-bib-0060] Olver, M. E. , and E. K. Riemer . 2021. “High‐Psychopathy Men With a History of Sexual Offending Have Protective Factors Too: But Are These Risk Relevant and Can They Change in Treatment?” Journal of Consulting and Clinical Psychology 89, no. 5: 406–420. 10.1037/ccp0000638. 33829820

[jopy70017-bib-0061] Patrick, C. J. 2010. Triarchic Psychopathy Measure (TriPM). PhenX Toolkit Online Assessment Catalog.

[jopy70017-bib-0062] Patrick, C. J. 2022. “Psychopathy: Current Knowledge and Future Directions.” Annual Review of Clinical Psychology 18, no. 1: 387–415. 10.1146/annurev-clinpsy-072720-012851. 35119947

[jopy70017-bib-0063] Patrick, C. J. , and L. E. Drislane . 2015. “Triarchic Model of Psychopathy: Origins, Operationalizations, and Observed Linkages With Personality and General Psychopathology.” Journal of Personality 83, no. 6: 627–643. 10.1111/jopy.12119. 25109906

[jopy70017-bib-0064] Patrick, C. J. , D. C. Fowles , and R. F. Krueger . 2009. “Triarchic Conceptualization of Psychopathy: Developmental Origins of Disinhibition, Boldness, and Meanness.” Development and Psychopathology 21, no. 3: 913–938. 10.1017/S0954579409000492. 19583890

[jopy70017-bib-0065] Paulhus, D. L. , C. S. Neumann , and R. D. Hare . 2016. Manual for the Self‐Report Psychopathy Scale. Multi‐Health Systems.

[jopy70017-bib-0066] Peterson, Z. D. , E. Janssen , D. Goodrich , J. D. Fortenberry , D. J. Hensel , and J. R. Heiman . 2018. “Child Sexual Abuse and Negative Affect as Shared Risk Factors for Sexual Aggression and Sexual HIV Risk Behavior in Heterosexual Men.” Archives of Sexual Behavior 47, no. 2: 465–480. 10.1007/s10508-017-1079-1. 29090393 PMC5775919

[jopy70017-bib-0068] Porter, S. , M. Woodworth , J. Earle , J. Drugge , and D. Boer . 2003. “Characteristics of Sexual Homicides Committed by Psychopathic and Nonpsychopathic Offenders.” Law and Human Behavior 27, no. 5: 459–470. 10.1023/a:1025461421791. 14593792

[jopy70017-bib-0069] Prentky, R. , and A. W. Burgess . 1990. “Rehabilitation of Child Molesters: A Cost‐Benefit Analysis.” American Journal of Orthopsychiatry 60, no. 1: 108–117. 10.1037/h0079197. 2106263

[jopy70017-bib-0070] Rousseeuw, P. J. 1990. “Robust Estimation and Identifying Outliers.” In Handbook of Statistical Methods for Engineers and Scientists. McGraw‐Hill Education.

[jopy70017-bib-0503] Roy, S. , C. Vize , K. Uzieblo , et al. 2021. “Triarchic or Septarchic?—Uncovering the Triarchic Psychopathy Measure's (TriPM) Structure.” Personality Disorders: Theory, Research, and Treatment 12, no. 1: 1–15. 10.1037/per0000392. 31971417

[jopy70017-bib-0071] RStudio Team . 2016. RStudio: bIntegrated Development for R [Computer Software]. RStudio, Inc. http://www.rstudio.com/.

[jopy70017-bib-0072] Sharpe, B. M. , K. L. Collison , D. R. Lynam , and J. D. Miller . 2021. “Does Machiavellianism Meaningfully Differ From Psychopathy? It Depends.” Behavioral Sciences & the Law 39, no. 5: 663–677. 10.1002/bsl.2538. 34636074

[jopy70017-bib-0073] Sleep, C. E. , B. Weiss , D. R. Lynam , and J. D. Miller . 2019. “An Examination of the Triarchic Model of Psychopathy's Nomological Network: A Meta‐Analytic Review.” Clinical Psychology Review 71: 1–26. 10.1016/j.cpr.2019.04.005. 31078055

[jopy70017-bib-0074] Tonidandel, S. , and J. M. LeBreton . 2015. “RWA Web: A Free, Comprehensive, Web‐Based, and User‐Friendly Tool for Relative Weight Analyses.” Journal of Business and Psychology 30, no. 2: 207–216. 10.1007/s10869-014-9351-z.

[jopy70017-bib-0075] Venables, N. C. , J. R. Hall , and C. J. Patrick . 2014. “Differentiating Psychopathy From Antisocial Personality Disorder: A Triarchic Model Perspective.” Psychological Medicine 44, no. 5: 1005–1013. 10.1017/S003329171300161X. 23834781

[jopy70017-bib-0076] Vevea, J. L. , and C. M. Woods . 2005. “Conducting Meta‐Analyses in R With the Metafor Package.” Psychological Methods 10, no. 4: 428–443. 10.1037/1082-989X.10.4.428. 16392998

[jopy70017-bib-0077] Viechtbauer, W. 2010. “Conducting Meta‐Analyses in R With the Metafor Package.” Journal of Statistical Software 36, no. 3: 1–48. 10.18637/jss.v036.i03.

[jopy70017-bib-0078] Vize, C. E. , K. L. Collison , J. D. Miller , and D. R. Lynam . 2019. “Using Bayesian Methods to Update and Expand the Meta‐Analytic Evidence of the Five‐Factor Model's Relation to Antisocial Behavior.” Clinical Psychology Review 67: 61–77. 10.1016/j.cpr.2018.09.001. 30292437

[jopy70017-bib-0079] Vize, C. E. , D. R. Lynam , K. L. Collison , and J. D. Miller . 2018. “Differences Among Dark Triad Components: A Meta‐Analytic Investigation.” Personality Disorders, Theory, Research, and Treatment 9, no. 2: 101–111. 10.1037/per0000222. 27736106

[jopy70017-bib-0080] Walters, G. D. 2003. “Predicting Criminal Justice Outcomes With the Psychopathy Checklist and Lifestyle Criminality Screening Form: A Meta‐Analytic Comparison.” Behavioral Sciences & the Law 21, no. 1: 89–102. 10.1002/bsl.519. 12579620

