# Supplementary material for: The Relation Between Psychopathy and Sexual Aggression: A Meta‐Analysis
Source: J Pers. 2025 Aug 31;94(3):487–506. doi: 10.1111/jopy.70017 (PMC13163617; doi:10.1111/jopy.70017)
Supplement: Supplementary file 1 — Data S1: jopy70017‐sup‐0001‐DataS1.docx. [file JOPY-94-487-s001.docx]

Supplementary Table 1

Characteristics of studies included in meta-analysis

| Citation | Sample Size | %  Male | %  White | Mean  Age | Gen sample  type | Psy Measure | SA measure | Sample's country  of origin |
| --- | --- | --- | --- | --- | --- | --- | --- | --- |
| Abbey et al. (2011) | 470 | 100 | 73 | 24 | 2 | SRP-III | SES-16-item version | US |
| Barbaree et al. (2001) | 215 | 100 | NA | 38 | 5 | PCL-R | SORAG, RRASOR, Static-99, MnSOST-R, MASORR | Canada |
| Beggs & Grace (2008) | 216 | 100 | NA | 41 | 5 | PCL-R | Static-99 | New Zealand |
| Bouffard et al. (2016) | 582 | 0 | 77 | 22 | 1 | LSRP | Self-reported SA hx | US |
| Bouffard & Miller (2022) | 481 | 100 | 76 | 22 | 1 | LSRP | Self-reported SA hx | US |
| Brassard et al. (2022) | 226 | 100 | NA | 34 | 6 | LSRP, French version | CTS2 sexual violence subscale | Canada |
| Brereton (2021) | 1132 | 100 | 44 | 20 | 1 | SRP-SF | SES-SF | US |
| Brown et al. (2022) | 4280 | 50 | 83 | 31 | 2 | SD3 | SFBI | Multiple |
| Brown et al. (2015) | 2514 | 100 | 73 | 31 | 3 | PCL-R | Criminal records | US |
| Camilleri & Quinsey (2009) | 115 | 100 | NA | NA | 3 | PCL-R | Criminal records | Canada |
| Caperton (2006) | 1983 | 100 | 48 | 45 | 5 | PCL-R | Static-99; MnSOST-R | US |
| Cardona et al. (2020) | 302 | 100 | 69 | 40 | 5 | PCL-R | SAG | US |
| Carton & Egan (2017) | 128 | 82 | 90 | NA | 2 | SD3 | CTS2S | UK |
| Chakhssi et al. (2013) | 66 | 100 | NA | 41 | 3 | PCL-R, Dutch version | Criminal records | Netherlands |
| Christopher et al. (2007) | 61 | 0 | 58 | NA | 3 | LSRP | Criminal records | US |
| Clounch (2009) | 583 | 100 | NA | NA | 5 | PCL:SV | Static-99 | US |
| Cohen & Galynker (2012) | 82 | NA | NA | NA | 6 | PCL-R | Criminal records | US |
| Costello et al. (2020) | 401 | 47 | 72 | 36 | 2 | TriPM, PPI-R | ISOS-PR | US |
| Costello et al. (2020) | 419 | 52 | 83 | 37 | 2 | TriPM, PPI-40 | ISOS-PR | US |
| Darjee (2019) | 51 | 100 | 100 | 26 | 5 | PCL-R | SeSaS | UK |
| DeGue & DiLillo (2004) | 304 | 100 | 88 | 21 | 1 | PPI-SF | SEQ | US |
| DeGue et al. (2010) | 369 | 100 | 66 | 32 | 3 | PPI-SF | SEQ | US |
| DeLisi et al. (2022) | 636 | 97 | 36 | 20 | 3 | PCL-R | Clinician ratings/DSM criteria | US |
| Di Francisco (2006) | 80 | 100 | 78 | 37 | 5 | PCL-R | SASI | US |
| Doolan (2017) | 46 | 100 | 65 | 35 | 2 | SRP-SF | SES-SFP | US |
| Fernandez & Marshall (2003) | 88 | 100 | 100 | 34 | 3 | PCL-R | Criminal records | Canada |
| Ferretti et al. (2021) | 190 | NA | NA | 45 | 3 | PCL-R, Italian version | Criminal records | Italy |
| Fischer (2008) | 10 | 100 | 80 | 53 | 5 | PCL-R | Sexual Sadism Criteria of Offenses from Dietz et al. (1990) | US |
| Garofalo et al. (2018) | 138 | 100 | NA | 43 | 6 | LSRP, Dutch version | Criminal records | Netherlands |
| Gonçalves et al. (2020) | 179 | 100 | NA | 39 | 5 | PCL-R | SeSaS; Static-99; SORAG; Clinician ratings/DSM criteria | Switzerland |
| Greenfield et al. (2021) | 287 | 100 | 31 | 37 | 3 | PCL-R | Criminal records; SeSaS | US |
| Häkkänen-Nyholm et al. (2009) | 676 | NA | NA | 35 | 5 | PCL-R | Criminal records | Finland |
| Hamburger (1995) | 242 | 100 | NA | 19 | 1 | SRP2 | CSS; SES; SEQ | US |
| Harris et al. (2007) | 512 | 100 | NA | 30 | 5 | PCL-R | Cormier-Lang score for adult sex offenses; clinical files | Canada |
| Heirigs (2020) | 320 | 41 | 75 | 37 | 3 | PPI-SF | Criminal records | US |
| Hertz et al. (2021) | 534 | 100 | NA | NA | 5 | PCL-R, German version | VRAG-R; Static-99; SORAG | Austria |
| Hill et al. (2012) | 166 | 100 | NA | 39 | 5 | PCL-R, German version | Static-99; SVR-20 | Germany |
| Hoffmann & Verona (2019) | 1199 | 37 | 69 | 21 | 1 | SRP-III | PSP | US |
| Hoffmann & Verona (2021) | 318 | 58 | 37 | 35 | 3 | PCL:SV | CTS2 sexual coercion subscale | US |
| Holt et al. (1999) | 41 | 100 | 39 | 31 | 3 | PCL-R | Clinician ratings/DSM criteria | US |
| Howard et al. (2012) | 100 | 100 | 89 | 35 | 6 | PCL-R | Criminal records | UK |
| Iyican & Babcock (2018) | 114 | 100 | 27 | 32 | 2 | PPI-SF | CTS2 sexual coercion subscale | US |
| Jabbour (2009) | 54 | NA | 81 | 40 | 5 | PPI | Criminal records | US |
| James, Higgs, & Proulx (unpublished) | 120 | 100 | 70 | 30 | 5 | PCL:SV | SeSaS; number of sexual homicides | France |
| Johansson et al. (2008) | 938 | 100 | NA | 38 | 2 | SRP-III | Sexual Coercion Scale | Finland |
| Jones (2011) | 39 | 100 | 38 | 30 | 6 | PCL:SV | Criminal records | US |
| Juodis et al. (2009) | 125 | 100 | NA | 30 | 3 | PCL-R | Criminal records | Canada |
| Kosson et al. (1997) | 378 | 100 | NA | NA | 1 | PCL:SV | SES | US |
| Kostiuk (2013) | 90 | 100 | 61 | 36 | 3 | SRP-III | SORAG; police records | Canada |
| Lalumière & Quinsey (1996) | 99 | 100 | NA | 23 | 1 | LSRP | SES | Canada |
| Lanaville (2018) | 385 | 53 | 85 | 35 | 2 | PPI-40 | SES | US |
| Langevin & Curnoe (2010) | 1695 | 100 | NA | 35 | 3 | PCL-R | Criminal records | Canada |
| Levenson & Morin (2006) | 450 | 100 | 56 | 41 | 5 | PCL-R | MnSOST-R; Static-99; criminal records; clinician ratings/DSM criteria | US |
| Lewing (2006) | 416 | 100 | 68 | 31 | 6 | LSRP | Criminal records | US |
| Lodi-Smith et al. (2014) | 585 | 41 | 88 | 24 | 2 | Dirty Dozen psychopathy subscale | MIDSA | US |
| Lord (2014) | 115 | 100 | 24 | 21 | 1 | SRP-III | SES; CTS2 | US |
| Lyons et al. (2022) | 208 | 38 | NA | 23 | 2 | LSRP | PSP | UK |
| March & Wagstaff (2017) | 240 | 28 | NA | 26 | 2 | LSRP | Explicit Image Scale | Australia |
| Monti (2019) | 214 | 100 | 81 | 35 | 2 | SRP-III | SES | US |
| Mouilso & Calhoun (2012) | 314 | 100 | 77 | 19 | 1 | SRP-III | SES | US |
| Muñoz Centifanti et al. (2016) | 164 | 44 | 85 | 22 | 2 | LSRP | PSP | UK |
| Navas et al. (2021) | 234 | 100 | NA | 43 | 6 | Dirty Dozen psychopathy subscale | Criminal records | Spain |
| Nielsen (2018) | 127 | 100 | 84 | NA | 5 | PCL-R | SORAG | US |
| Nuckols (2011) | 26 | 100 | NA | 37 | 3 | PCL:SV | Criminal records | US |
| Parent et al. (2011) | 503 | 100 | 91 | 33 | 5 | PCL-R | SORAG, RRASOR, Static-99, Static-2002, MnSOST-R, SVR-20, RM2000/S | US |
| Petruccelli et al. (2017) | 79 | 100 | NA | NA | 3 | PCL-R, Italian version | Criminal records | Italy |
| Porter et al. (2000) | 329 | 100 | 71 | 44 | 5 | PCL-R | Criminal records | Canada |
| Robertson & Knight (2014) | 314 | 100 | 67 | 39 | 5 | PCL-R | MIDSA Sexual Sadism Scale | US |
| Robertson & Knight (2014) | 599 | 100 | 91 | 36 | 5 | PCL-R | MTC: CM3 and R3 sadism criteria categories | US |
| Rosa et al. (2022) | 1632 | 0 | NA | 22 | 1 | YPI-S, Portuguese version | SABS | Portugal |
| Sánchez-Medina et al. (2020) | 374 | NA | NA | NA | 1 | Dirty Dozen psychopathy subscale | Items measuring sexual cyberbullying/  harassment | Spain |
| Savage (2015) | 91 | 0 | 88 | 21 | 1 | SRP-III | PSP | Canada |
| Schimmenti et al. (2014) | 87 | 100 | 100 | 44 | 3 | PCL-R, Italian version | Criminal records | Italy |
| Sewall & Olver (2019) | 302 | 100 | 63 | 33 | 5 | PCL-R | VRS-SO; SORAG no PCL-R incl | Canada |
| Skovran et al., (2010) | 199 | 100 | 80 | 34 | 3 | PCL-R | Criminal records | US |
| Sloan (2003) | 68 | 100 | 84 | 22 | 1 | PPI | SES | US |
| Sohn et al. (2022) | 451 | 100 | 0 | 39 | 3 | PCL-R, Korean version | Criminal records | South Korea |
| Stewart (unpublished) | 865 | 20 | 31 | 21 | 1 | SD3-P | SES; SCIRS | US |
| Stoll et al. (2019) | 64 | 100 | NA | NA | 6 | PCL:SV | MSI Child Molest subscale | Switzerland |
| Strassberg et al. (2012) | 74 | 100 | NA | 32 | 6 | PPI | Criminal records | US |
| Strassberg et al. (2012) | 79 | 100 | NA | 32 | 6 | PPI | Criminal records | US |
| Tang et al. (2020) | 856 | 1 | NA | 27 | 2 | German Naughty Nine psychopathy subscale | Items measuring sexual cyberbullying/  harassment | Germany |
| Tristan (2007) | 1739 | 100 | 84 | NA | 5 | PCL-R | Static-99; MnSOST-R | US |
| Tsao & Chu (2021) | 134 | 100 | NA | 24 | 5 | PCL-R | Static-99R; SVR-20 v2 | Singapore |
| Tülü & Erden (2014) | 206 | 100 | NA | 33 | 6 | LSRP | Criminal records | Turkey |
| Verona, E. (unpublished) | 298 | 100 | 64 | 20 | 1 | SRP-III | PSP | US |
| Verona, E. (unpublished) | 192 | 53 | 61 | 31 | 2 | SRP-III | CTS Sexual IPV perpetration | US |
| Vess et al. (2004) | 968 | 100 | 49 | 40 | 5 | PCL-R | Criminal records | US |
| Voller (2010) | 521 | 100 | 82 | 20 | 1 | PPI-R | SES | US |
| Warkentin (2008) | 514 | 100 | 91 | NA | 1 | LSRP | SES | US |
| Warkentin & Gidycz (2007) | 297 | 100 | 94 | 19 | 1 | LSRP | SES | US |
| Watts et al. (2017) | 608 | 27 | 41 | 19 | 1 | TriPM, PPI-R, LSRP | Freund's Paraphilia Scales | US |
| Wegrzyn et al. (2017) | 62 | 100 | NA | 42 | 6 | PPI-R | Criminal records | Germany |
| Williams et al. (2009) | 88 | 100 | 58 | 20 | 1 | SRP-III | MASA | Canada |
| Woehrle et al. (2022) | 239 | 100 | NA | 36 | 3 | PCL-R, German version | Criminal records | Germany |
| Woodworth et al. (2013) | 139 | 100 | NA | 44 | 5 | PCL-R | Criminal records | Canada |
| Yoon (2010) | 46 | 100 | NA | NA | 1 | PPI | MASA | US |
| Young et al. (2010) | 120 | 100 | 43 | 33 | 3 | PCL-R | Criminal records | US |

*Note*. SA = sexual aggression; IPV = Intimate Partner Violence; DSM = Diagnostic and Statistical Manual of Mental Disorders; SRP = Self-Report Psychopathy Scale; SRP-SF = Self-Report Psychopathy Scale-Short Form; PCL-R = Psychopathy Checklist-Revised; LSRP = Levenson Self-Report Psychopathy scale; SD3 = Short Dark Triad; PCL:SV = Psychopathy Checklist: Screening Version; TriPM = Triarchic Psychopathy Measure; PPI = Psychopathic Personality Inventory; PPI-R = Psychopathic Personality Inventory-Revised; PPI-40 = Psychopathic Personality Inventory 40-item version; PPI-SF = Psychopathic Personality Inventory-Short Form; YPI-S = Youth Psychopathic Traits Inventory; SES = Sexual Experience Survey; SES-SFP = Sexual Experience Survey-Short Form Perpetration; SORAG = Sex Offender Risk Appraisal Guide; RRASOR = Rapid Risk Assessment for Sexual Offense Recidivism; MnSOST-R = Minnesota Sex Offender Screening Tool-Revised; MASORR = Multifactorial Assessment of Sex Offender Risk for Recidivism; CTS = Conflict Tactics Scale; CTS2S = Revised Conflict Tactics Scale Short Form; SFBI = Sexual Fantasies and Behaviors Inventory; SAG = physical aggression within sexual crimes; ISOS-PR = Interpersonal Sexual Objectification Scale-Perpetration Revised; SeSaS = Severe Sexual Sadism Scale; SEQ = Sexual Experiences Questionnaire; SASI = Sexual Aggression Severity Inventory; CSS = Coercive Sexuality Scale; VRAG-R = Violence Risk Appraisal Guide-Revised; SVR-20 = Sexual Violence Risk-20; PSP = Postrefusal Sexual Persistence Scale; MIDSA = Multidimensional Inventory of Development, Sex, and Aggression; RM2000/S = Risk Matrix 2000/Sexual Offending; MTC: CM3 and R3 sadism criteria categories = Massachusetts Treatment Center: Child Molester Typology, Version 3, and Rapist Typology, Version 3; SABS = Sexually Aggressive Behaviors Scale; VRS-SO = Violence Risk Scale: Sexual Offender Version; SCIRS = Sexual Coercion in Intimate Relationships Scale; MSI = Multiphasic Sexual Inventory; MASA = Multidimensional Assessment of Sexual Aggression.

Supplementary Table 2

Relative weights of the triarchic model of psychopathy in the current study’s included measures

|  | Correlations | | | Raw Relative Weights | | | | Rescaled Relative Weights | | | |  | |
| --- | --- | --- | --- | --- | --- | --- | --- | --- | --- | --- | --- | --- | --- |
| Scale | B | M | D | | B | M | D | | B | M | D | | R-Squared |
| YPI-S^1^ | 0.32 | 0.44 | 0.38 | | 0.09 | 0.10 | 0.10 | | 30.86 | 35.25 | 33.89 | | 0.29 |
| SRP-III^2^ | 0.35 | 0.59 | 0.48 | | 0.10 | 0.22 | 0.16 | | 21.56 | 44.63 | 33.82 | | 0.49 |
| SRP-SF^2^ | 0.35 | 0.59 | 0.48 | | 0.10 | 0.22 | 0.16 | | 21.56 | 44.63 | 33.82 | | 0.49 |
| SRP-II^2^ | 0.35 | 0.59 | 0.48 | | 0.10 | 0.22 | 0.16 | | 21.56 | 44.63 | 33.82 | | 0.49 |
| SD3^3^ | 0.26 | 0.70 | 0.59 | | 0.05 | 0.31 | 0.21 | | 8.92 | 53.94 | 37.14 | | 0.58 |
| PPI^2^ | 0.52 | 0.57 | 0.49 | | 0.25 | 0.18 | 0.19 | | 40.45 | 28.69 | 30.86 | | 0.62 |
| PPI-R^4^ | 0.58 | 0.64 | 0.48 | | 0.29 | 0.23 | 0.15 | | 43.27 | 34.06 | 22.67 | | 0.67 |
| PPI-40^4^ | 0.58 | 0.64 | 0.48 | | 0.29 | 0.23 | 0.15 | | 43.27 | 34.06 | 22.67 | | 0.67 |
| PPI-SF^5^ | 0.49 | 0.63 | 0.37 | | 0.20 | 0.29 | 0.09 | | 34.43 | 50.06 | 15.50 | | 0.57 |
| PCL-R^6^ | 0.28 | 0.39 | 0.33 | | 0.07 | 0.08 | 0.07 | | 32.06 | 36.10 | 31.84 | | 0.22 |
| PCL:SV^7^ | 0.06 | 0.27 | 0.21 | | 0.00 | 0.05 | 0.03 | | 2.47 | 65.66 | 31.87 | | 0.08 |
| LSRP^2^ | 0.00 | 0.57 | 0.61 | | 0.01 | 0.22 | 0.26 | | 1.09 | 45.23 | 53.68 | | 0.48 |
| TriPM Total^8^ | 0.65 | 0.84 | 0.64 | | 0.33 | 0.41 | 0.27 | | 32.70 | 40.73 | 26.57 | | 1.01 |
| TriPM Boldness^8^ | 1.00 | 0.29 | -0.02 | | 0.95 | 0.04 | 0.00 | | 95.22 | 4.44 | 0.33 | | 1.00 |
| TriPM Meanness^8^ | 0.29 | 1.00 | 0.49 | | 0.04 | 0.83 | 0.12 | | 4.44 | 83.32 | 12.24 | | 1.00 |
| TriPM Disinhibition^8^ | -0.02 | 0.49 | 1.00 | | 0.00 | 0.12 | 0.87 | | 0.33 | 12.24 | 87.43 | | 1.00 |
| *Note*. B = boldness; M = meanness; D = disinhibition; YPI-S = Youth Psychopathic Traits Inventory; SRP = Self-Report Psychopathy Scale; SRP-SF = Self-Report Psychopathy Scale-Short Form; SD3 = Short Dark Triad; PPI = Psychopathic Personality Inventory; PPI-R = Psychopathic Personality Inventory-Revised; PPI-40 = Psychopathic Personality Inventory 40-item version; PPI-SF = Psychopathic Personality Inventory-Short Form; PCL-R = Psychopathy Checklist-Revised; PCL:SV = Psychopathy Checklist: Screening Version; LSRP = Levenson Self-Report Psychopathy scale; TriPM = Triarchic Psychopathy Measure. Citations for source of correlations used for relative weights calculations: 1. Fanti et al., 2016; 2. Drislane et al. (2014); 3. Stanton et al. (2021); 4. van Dongen et al. (2017); 5. Stanley et al. (2013); 6. Venables et al. (2014); 7. Sellbom et al. (2018); 8. Sharpe et al. (2021). TriPM correlations from Sharpe et al. (2021) were only used to calculate a measure’s relative weights when that measure’s study did not provide its own TriPM correlations. | | | | | | | | | | | | | |

**Appendix A. References for Studies Included in Meta-Analysis**

Abbey A, Jacques-Tiura AJ, & LeBreton JM. (2011). Risk factors for sexual aggression in young men: An expansion of the confluence model. *Aggress Behav*, *37*(5), 450–464. https://doi.org/10.1002/ab.20399

Barbaree, H. E., Seto, M. C., Langton, C. M., & Peacock, E. J. (2001). Evaluating the predictive accuracy of six risk assessment instruments for adult sex offenders. *Criminal Justice and Behavior*, *28*(4), 490–521. https://doi.org/10.1177/009385480102800406

Beggs, S. M., & Grace, R. C. (2008). Psychopathy, Intelligence, and Recidivism in Child Molesters: Evidence of an Interaction Effect. *Criminal Justice and Behavior*, *35*(6), 683–695. https://doi.org/10.1177/0093854808314786

Bouffard, J. A., & Miller, H. A. (2021). Exploring rape myth acceptance and psychopathy as predictors of sexual coercion. *Journal of Aggression, Maltreatment & Trauma*. https://doi.org/10.1080/10926771.2021.1994501

Bouffard JA, Bouffard LA, & Miller HA. (2016). Examining the Correlates of Women’s Use of Sexual Coercion: Proposing an Explanatory Model. *J Interpers Violence*, *31*(13), 2360–2382. https://doi.org/10.1177/0886260515575609

Brassard, A., Gagnon, C., Claing, A., Dugal, C., Savard, C., & Péloquin, K. (2022). Can romantic attachment and psychopathy concomitantly explain the forms and severity of perpetrated intimate partner violence in men seeking treatment? *Partner Abuse*, *13*(1), 123–143. https://doi.org/10.1891/PA-2021-0008

Brereton, A. L. (2021). *Attachment and campus sexual misconduct: The mediating roles of psychopathy and empathy* (2020-67315-194; Issues 2-B) [ProQuest Information & Learning].

Brown A, Barker ED, & Rahman Q. (2022). Psychological and Developmental Correlates of Paraphilic and Normophilic Sexual Interests. *Sex Abuse*, 10790632221120013. https://doi.org/10.1177/10790632221120013

Brown, A. R., Dargis, M. A., Mattern, A. C., Tsonis, M. A., & Newman, J. P. (2015). Elevated Psychopathy Scores Among Mixed Sexual Offenders: Replication and Extension. *Criminal Justice and Behavior*, *42*(10), 1032–1044. https://doi.org/10.1177/0093854815575389

Camilleri JA & Quinsey VL. (2009). Individual differences in the propensity for partner sexual coercion. *Sex Abuse*, *21*(1), 111–129. https://doi.org/10.1177/1079063208327237

Caperton, J. D. (2006). *Predicting recidivism among sex offenders: Utility of the static-99, Minnesota Sex Offender Screening Tool-Revised, and Psychopathy Checklist-Revised* (2006-99006-105; Issues 9-B) [ProQuest Information & Learning].

Cardona N, Berman AK, Sims-Knight JE, & Knight RA. (2020). Covariates of the Severity of Aggression in Sexual Crimes: Psychopathy and Borderline Characteristics. *Sex Abuse*, *32*(2), 154–178. https://doi.org/10.1177/1079063218807485

Carton, H., & Egan, V. (2017). The dark triad and intimate partner violence. *Personality and Individual Differences*, *105*, 84–88. https://doi.org/10.1016/j.paid.2016.09.040

Chakhssi F, de Ruiter C, & Bernstein DP. (2013). Early maladaptive cognitive schemas in child sexual offenders compared with sexual offenders against adults and nonsexual violent offenders: An exploratory study. *J Sex Med*, *10*(9), 2201–2210. https://doi.org/10.1111/jsm.12171

Christopher, K., Lutz-Zois, C. J., & Reinhardt, A. R. (2007). Female sexual-offenders: Personality pathology as a mediator of the relationship between childhood sexual abuse history and sexual abuse. *Child Abuse & Neglect*, *31*(8), 871–883. https://doi.org/10.1016/j.chiabu.2007.02.006

Clounch, K. L. (2009). *Sex offender assessment: Clinical utility and predictive validity* (2009-99040-367; Issues 8-B) [ProQuest Information & Learning].

Cohen LJ & Galynker I. (2012). Identifying psychological traits potentially subserving aberrant motivation or inhibitory failure in pedophilic behavior. *Isr J Psychiatry Relat Sci*, *49*(4), 280–290.

Costello TH, Watts AL, Murphy BA, & Lilienfeld SO. (2020). Extending the nomological network of sexual objectification to psychopathic and allied personality traits. *Personal Disord*, *11*(4), 237–248. https://doi.org/10.1037/per0000377

Darjee R. (2019). Sexual Sadism and Psychopathy in Sexual Homicide Offenders: An Exploration of Their Associates in a Clinical Sample. *Int J Offender Ther Comp Criminol*, *63*(9), 1738–1765. https://doi.org/10.1177/0306624X19836872

DeGue, S., & DiLillo, D. (2004). Understanding Perpetrators of Nonphysical Sexual Coercion: Characteristics of Those Who Cross the Line. *Violence and Victims*, *19*(6), 673–688. https://doi.org/10.1891/vivi.19.6.673.66345

DeGue, S., DiLillo, D., & Scalora, M. (2010). Are All Perpetrators Alike? Comparing Risk Factors for Sexual Coercion and Aggression. *Sexual Abuse: A Journal of Research and Treatment*, *22*(4), 402–426. https://doi.org/10.1177/1079063210372140

DeLisi M, Peters DJ, Hochstetler A, Butler HD, & Vaughn MG. (2022). Psychopathy among condemned capital murderers. *J Forensic Sci*. https://doi.org/10.1111/1556-4029.15188

Di Francisco, M. (2006). *Psychopathy, negative emotions of anger and depression, and causal attributions: Relation to sexual aggression* (2006-99018-312; Issues 3-B) [ProQuest Information & Learning].

Doolan, P. L. (2017). *Personality, sexual fantasy, and sexually deviant behavior in a community male sample* (2017-10863-093; Issues 5-B(E)) [ProQuest Information & Learning].

Fernandez, Y. M., & Marshall, W. L. (2003). Victim Empathy, Social Self-Esteem, and Psychopathy in Rapists. *Sexual Abuse*, *15*(1), 11–26. https://doi.org/10.1177/107906320301500102

Ferretti, F., Pozza, A., Carabellese, F., Schimmenti, A., Santoro, G., Mandarelli, G., Gualtieri, G., Carabellese, F., Catanesi, R., & Coluccia, A. (2021). Non-intimate Relationships and Psychopathic Interpersonal and Affective Deficits as Risk Factors for Criminal Career: A Comparison Between Sex Offenders and Other Offenders. *Frontiers in Psychology*, *12*, 600370. https://doi.org/10.3389/fpsyg.2021.600370

Fischer, E. G. J. (2008). *Profiling sexually violent predators: A study to identify psychopathy and sexual sadism as baseline psychopathologies in predatory sexual serial offenders* (2008-99140-361; Issues 1-B) [ProQuest Information & Learning].

Garofalo, C., Bogaerts, S., & Denissen, J. J. A. (2018). Personality functioning and psychopathic traits in child molesters and violent offenders. *Journal of Criminal Justice*, *55*, 80–87. https://doi.org/10.1016/j.jcrimjus.2018.02.003

Gonçalves, L. C., Rossegger, A., Gerth, J., Singh, J. P., & Endrass, J. (2020). Sexual sadism among sex offenders in Switzerland. *Sexual Abuse: Journal of Research and Treatment*, *32*(1), 79–100. https://doi.org/10.1177/1079063218800473

Greenfield, D. N., Cazala, F., Carre, J., Mitchell-Somoza, A., Decety, J., Thornton, D., Kiehl, K. A., & Harenski, C. L. (2021). Emotional intelligence in incarcerated sexual offenders with sexual sadism. *Journal of Sexual Aggression*. https://doi.org/10.1080/13552600.2021.2015469

Häkkänen-Nyholm H, Repo-Tiihonen E, Lindberg N, Salenius S, & Weizmann-Henelius G. (2009). Finnish sexual homicides: Offence and offender characteristics. *Forensic Sci Int*, *188*(1–3), 125–130. https://doi.org/10.1016/j.forsciint.2009.03.030

Hamburger, M. E. (1995). *Assessing the validity of a multidimensional model of sexual coercion in college men* (1995-95021-074; Issues 5-B) [ProQuest Information & Learning].

Harris, G. T., Rice, M. E., Hilton, N. Z., Lalumiére, M. L., & Quinsey, V. L. (2007). Coercive and Precocious Sexuality as a Fundamental Aspect of Psychopathy. *Journal of Personality Disorders*, *21*(1), 1–27. https://doi.org/10.1521/pedi.2007.21.1.1

Heirigs, M. H. (2020). *Psychopathy, adverse childhood experiences, and antisocial behavior* [Graduate Theses and Dissertations, Iowa State University]. https://lib.dr.iastate.edu/etd/17932

Hertz, P. G., Eher, R., Etzler, S., & Rettenberger, M. (2021). Cross-validation of the revised

version of the Violence Risk Appraisal Guide (VRAG-R) in a sample of individuals

convicted of sexual offenses. *Sexual Abuse: Journal of Research and Treatment*, *33*(1),

63–87.

Hill, A., Rettenberger, M., Habermann, N., Berner, W., Eher, R., & Briken, P. (2012). The utility of risk assessment instruments for the prediction of recidivism in sexual homicide perpetrators. *Journal of Interpersonal Violence*, *27*(18), 3553–3578. https://doi.org/10.1177/0886260512447570

Hoffmann AM & Verona E. (2019). Psychopathic traits, gender, and motivations for sex: Putative paths to sexual coercion. *Aggress Behav*, *45*(5), 527–536. https://doi.org/10.1002/ab.21841

Hoffmann AM & Verona E. (2021). Psychopathic Traits and Sexual Coercion Against Relationship Partners in Men and Women. *J Interpers Violence*, *36*(3–4), NP1788-1809NP. https://doi.org/10.1177/0886260518754873

Holt SE, Meloy JR, & Strack S. (1999). Sadism and psychopathy in violent and sexually violent offenders. *J Am Acad Psychiatry Law*, *27*(1), 23–32.

Howard, R., Khalifa, N., Duggan, C., & Lumsden, J. (2012). Are patients deemed ‘dangerous and severely personality disordered’ different from other personality disordered patients detained in forensic settings? *Criminal Behaviour and Mental Health*, *22*(1), 65–78. https://doi.org/10.1002/cbm.827

Iyican, S., & Babcock, J. C. (2018). The Relation Between the Two Factors of Psychopathy and Intimate Partner Aggression. *Journal of Aggression, Maltreatment & Trauma*, *27*(2), 119–130. https://doi.org/10.1080/10926771.2017.1334020

Jabbour, A. (2009). *Instrumentality, reactivity and psychopathy in sexual offenses against children: An exploratory analysis* (2010-99100-382; Issues 11-B) [ProQuest Information & Learning].

James, J., Higgs, T., & Proulx, J. (unpublished)

Johansson A, Santtila P, Harlaar N, von der Pahlen B, Witting K, Algars M, Alanko K, Jern P, Varjonen M, & Sandnabba NK. (2008). Genetic effects on male sexual coercion. *Aggress Behav*, *34*(2), 190–202. https://doi.org/10.1002/ab.20230

Jones, J. L. (2011). *Attachment, empathy and psychopathy in a sex offending sample* (2011-99100-422; Issues 11-B) [ProQuest Information & Learning].

Juodis, M., Woodworth, M., Porter, S., & Ten Brinke, L. (2009). Partners in crime: A comparison of individual and multi-perpetrator homicides. *Criminal Justice and Behavior*, *36*(8), 824–839. https://doi.org/10.1177/0093854809337822

Kosson, D. S., Kelly, J. C., & White, J. W. (1997). Psychopathy-Related Traits Predict Self-Reported Sexual Aggression Among College Men. *Journal of Interpersonal Violence*, *12*(2), 241–254. https://doi.org/10.1177/088626097012002006

Kostiuk, N. E. (2013). *Implicit and explicit self-esteem, narcissism, risk, and psychopathy in a forensic population* (2013-99200-377; Issues 4-B(E)) [ProQuest Information & Learning].

Lalumière, M. L., & Quinsey, V. L. (1996). Sexual deviance, antisociality, mating effort, and the use of sexually coercive behaviors. *Personality and Individual Differences*, *21*(1), 33–48. https://doi.org/10.1016/0191-8869(96)00059-1

Lanaville, D. Y. (2018). *Predicting adult sexual aggression in persons exposed to childhood sexual abuse: The moderating effects of general intelligence, emotional intelligence, and psychopathic deviancy* (2017-54456-136; Issues 1-B(E)) [ProQuest Information & Learning].

Langevin, R., & Curnoe, S. (2010). A comparison of psychopathy, attention deficit hyperactivity disorder, and brain dysfunction among sex offenders. *Journal of Forensic Psychology Practice*, *10*(3), 177–200. https://doi.org/10.1080/15228930903550624

Levenson, J. S., & Morin, J. W. (2006). Factors predicting selection of sexually violent predators for civil commitment. *International Journal of Offender Therapy and Comparative Criminology*, *50*(6), 609–629. https://doi.org/10.1177/0306624X06287644

Lewing, C. A. (2006). *Psychopathy as a moderator of the relationship between psychological reactance and sexual assault acceptance and perpetration among incarcerated and non-incarcerated males* (2006-99016-171; Issues 2-B) [ProQuest Information & Learning].

Lodi-Smith, J., Shepard, K., & Wagner, S. (2014). Personality and sexually deviant behavior. *Personality and Individual Differences*, *70*, 39–44. https://doi.org/10.1016/j.paid.2014.06.012

Lord, S. (2014). *Use of the self-report psychopathy scale iii facet scores in predicting dating violence and sexual aggression* (2014-99120-065; Issues 12-B(E)) [ProQuest Information & Learning].

Lyons M, Houghton E, Brewer G, & O’Brien F. (2022). The Dark Triad and Sexual Assertiveness Predict Sexual Coercion Differently in Men and Women. *J Interpers Violence*, *37*(7–8), NP4889–NP4904. https://doi.org/10.1177/0886260520922346

March, E., & Wagstaff, D. L. (2017). Sending Nudes: Sex, Self-Rated Mate Value, and Trait Machiavellianism Predict Sending Unsolicited Explicit Images. *Frontiers in Psychology*, *8*, 2210. https://doi.org/10.3389/fpsyg.2017.02210

Monti, E. (2019). *Will my voice tell on me? Assessment of victim vulnerability from voice* (2019-41129-145; Issues 6-B(E)) [ProQuest Information & Learning].

Mouilso, E. R., & Calhoun, K. S. (2012). A mediation model of the role of sociosexuality in the associations between narcissism, psychopathy, and sexual aggression. *Psychology of Violence*, *2*(1), 16–27. https://doi.org/10.1037/a0026217

Muñoz Centifanti LC, Thomson ND, & Kwok AH. (2016). Identifying the Manipulative Mating Methods Associated With Psychopathic Traits and BPD Features. *J Pers Disord*, *30*(6), 721–741. https://doi.org/10.1521/pedi_2015_29_225

Navas MP, Maneiro L, Cutrín O, Gómez-Fraguela JA, & Sobral J. (2021). Sexism, Moral Disengagement, and Dark Triad Traits on Perpetrators of Sexual Violence Against Women and Community Men. *Sex Abuse*, 10790632211051689. https://doi.org/10.1177/10790632211051689

Nielsen, L. (2018). *Differences in personality and psychopathy among probation eligible sexual offenders* (2018-09134-081; Issues 3-B(E)) [ProQuest Information & Learning].

Nuckols, A. E. (2011). *The roles of paternal attachment and psychopathy in the cycle of sexual abuse: Examining sex offenders and non-sex offenders* (2011-99100-423; Issues 11-B) [ProQuest Information & Learning].

Parent, G., Guay, J.-P., & Knight, R. A. (2011). An Assessment of Long-Term Risk of Recidivism By Adult Sex Offenders: One Size Doesn’t Fit All. *Criminal Justice and Behavior*, *38*(2), 188–209. https://doi.org/10.1177/0093854810388238

Petruccelli I, Barbaranelli C, Costantino V, Gherardini A, Grilli S, Craparo G, & D’Urso G. (2017). Moral Disengagement and Psychopathy: A Study on Offenders in Italian Jails. *Psychiatr Psychol Law*, *24*(5), 670–681. https://doi.org/10.1080/13218719.2017.1291291

Porter, S., Fairweather, D., Drugge, J., Hervé, H., Birt, A., & Boer, D. P. (2000). Profiles of Psychopathy in Incarcerated Sexual Offenders. *Criminal Justice and Behavior*, *27*(2), 216–233. https://doi.org/10.1177/0093854800027002005

Robertson CA & Knight RA. (2014). Relating sexual sadism and psychopathy to one another, non-sexual violence, and sexual crime behaviors. *Aggress Behav*, *40*(1), 12–23. https://doi.org/10.1002/ab.21505

Rosa PJ, Brazão N, & Carvalho J. (2022). Psychometric Properties of the Sexually Aggressive Behaviors Scale: Factor Structure, Reliability, and Construct Validity in a Sample of Portuguese Female College Students. *Int J Offender Ther Comp Criminol*, 306624X221113535. https://doi.org/10.1177/0306624X221113535

Sánchez-Medina, A. J., Galván-Sánchez, I., & Fernández-Monroy, M. (2020). Applying artificial intelligence to explore sexual cyberbullying behaviour. *Heliyon*, *6*(1), e03218. https://doi.org/10.1016/j.heliyon.2020.e03218

Savage, M. (2015). *The relationships among psychopathic traits and risky sexual behaviors, sexual coercion, and aggression in a female college sample* (2015-99100-284; Issues 11-B(E)) [ProQuest Information & Learning].

Schimmenti A, Passanisi A, & Caretti V. (2014). Interpersonal and affective traits of psychopathy in child sexual abusers: Evidence from a pilot study sample of Italian offenders. *J Child Sex Abus*, *23*(7), 853–860. https://doi.org/10.1080/10538712.2014.938210

Sewall LA & Olver ME. (2019). Psychopathy and treatment outcome: Results from a sexual violence reduction program. *Personal Disord*, *10*(1), 59–69. https://doi.org/10.1037/per0000297

Skovran, L. C., Huss, M. T., & Scalora, M. J. (2010). Sexual fantasies and sensation seeking among psychopathic sexual offenders. *Psychology, Crime & Law*, *16*(7), 617–629. https://doi.org/10.1080/10683160902998025

Sloan, L. G. Jr. (2003). *Sexual aggression and psychopathy: An examination of psychopathy as a moderating variable in sexual aggression* (2003-95010-084; Issues 11-B) [ProQuest Information & Learning]. https://login.ezproxy.lib.purdue.edu/login?url=https://search.ebscohost.com/login.aspx?direct=true&db=psyh&AN=2003-95010-084&site=ehost-live

Sohn JS, Reyes NC, & Kim H. (2022). Interpersonal and Affective Facets and Items of the Psychopathy Checklist-Revised (PCL-R) in Predicting Child Sex Offending. *J Interpers Violence*, *37*(9–10), NP6720–NP6732. https://doi.org/10.1177/0886260520958411

Stewart, R. (unpublished)

Stoll CB, Boillat C, Pflueger MO, Graf M, & Rosburg T. (2019). Psychopathy, Neuroticism, and Abusive Behavior in Low Risk Child Sex Offenders. *J Child Sex Abus*, *28*(8), 990–1006. https://doi.org/10.1080/10538712.2019.1630880

Strassberg DS, Eastvold A, Wilson Kenney J, & Suchy Y. (2012). Psychopathy among pedophilic and nonpedophilic child molesters. *Child Abuse Negl*, *36*(4), 379–382. https://doi.org/10.1016/j.chiabu.2011.09.018

Tang WY, Reer F, & Quandt T. (2020). Investigating sexual harassment in online video games: How personality and context factors are related to toxic sexual behaviors against fellow players. *Aggress Behav*, *46*(1), 127–135. https://doi.org/10.1002/ab.21873

Tristan, L. (2007). *Psychopathy factors and degree of forcefulness in sex-offenders: Implications for current risk-assessment practices* (2007-99018-330; Issues 3-B) [ProQuest Information & Learning].

Tsao IT & Chu CM. (2021). An Exploratory Study of Recidivism Risk Assessment Instruments for Individuals Convicted of Sexual Offenses in Singapore. *Sex Abuse*, *33*(2), 157–175. https://doi.org/10.1177/1079063219884575

Tulu, I. A., & Erden, H. G. (2013). Crime Analysis About Sex Offenders in Turkey: Rapists’ Psychological Profiling, Cognitive Distortions and Psychopathy. *Turkish Journal of Psychiatry*. https://doi.org/10.5080/u7070

Verona, E. (two unpublished studies)

Vess, J., Murphy, C., & Arkowitz, S. (2004). Clinical and demographic differences between sexually violent predators and other commitment types in a state forensic hospital. *Journal of Forensic Psychiatry & Psychology*, *15*(4), 669–681. https://doi.org/10.1080/14789940410001731795

Voller, E. K. (2010). *Personality variables in rape and sexual assault perpetration by college men: Psychopathy and the Five-Factor model* (2010-99160-461; Issues 2-B) [ProQuest Information & Learning].

Warkentin, J. B. (2008). *Dating violence and sexual assault among college men: Co-occurrence, predictors, and differentiating factors* (2008-99160-432; Issues 2-B) [ProQuest Information & Learning].

Warkentin, J. B., & Gidycz, C. A. (2007). The use and acceptance of sexually aggressive tactics in college men. *Journal of Interpersonal Violence*, *22*(7), 829–850. https://doi.org/10.1177/0886260507301793

Watts, A. L., Nagel, M. G., Latzman, R. D., & Lilienfeld, S. O. (2019). Personality Disorder Features and Paraphilic Interests Among Undergraduates: Differential Relations and Potential Antecedents. *Journal of Personality Disorders*, *33*(1), 22–48. https://doi.org/10.1521/pedi_2017_31_327

Wegrzyn M, Westphal S, & Kissler J. (2017). In your face: The biased judgement of fear-anger expressions in violent offenders. *BMC Psychol*, *5*(1), 16. https://doi.org/10.1186/s40359-017-0186-z

Williams, K. M., Cooper, B. S., Howell, T. M., Yuille, J. C., & Paulhus, D. L. (2009). Inferring sexually deviant behavior from corresponding fantasies: The role of personality and pornography consumption. *Criminal Justice and Behavior*, *36*(2), 198–222. https://doi.org/10.1177/0093854808327277

Woehrle, L., Retz-Junginger, P., Retz, W., & Barra, S. (2022). The Maltreatment–Aggression Link among Prosecuted Males: What about Psychopathy? *International Journal of Environmental Research and Public Health*, *19*(15), 9584. https://doi.org/10.3390/ijerph19159584

Woodworth M, Freimuth T, Hutton EL, Carpenter T, Agar AD, & Logan M. (2013). High-risk sexual offenders: An examination of sexual fantasy, sexual paraphilia, psychopathy, and offence characteristics. *Int J Law Psychiatry*, *36*(2), 144–156. https://doi.org/10.1016/j.ijlp.2013.01.007

Yoon, J. (2010). *Psychopathy in sexual coercion against women: The role of emotion and attention* (2010-99040-252; Issues 8-B) [ProQuest Information & Learning].

Young MH, Justice JV, & Edberg P. (2010). Sexual offenders in prison psychiatric treatment: A biopsychosocial description. *Int J Offender Ther Comp Criminol*, *54*(1), 92–112. https://doi.org/10.1177/0306624X08322373

**Appendix B. Studies Used to Derive Triarchic Psychopathy Relative Weights**

Drislane, L. E., Patrick, C. J., & Arsal, G. (2014). Clarifying the content coverage of differing psychopathy inventories through reference to the Triarchic Psychopathy Measure. *Psychological Assessment*, *26*(2), 350–362. https://doi.org/10.1037/a0035152

Fanti, K. A., Kyranides, M. N., Drislane, L. E., Colins, O. F., & Andershed, H. (2016). Validation of the Greek Cypriot Translation of the Triarchic Psychopathy Measure. *Journal of Personality Assessment*, *98*(2), 146–154. https://doi.org/10.1080/00223891.2015.1077452

Sellbom, M., Laurinavičius, A., Ustinavičiūtė, L., & Laurinaitytė, I. (2018). The Triarchic Psychopathy Measure: An examination in a Lithuanian inmate sample. *Psychological Assessment*, *30*(7), e10–e20. https://doi.org/10.1037/pas0000603

Sharpe, B. M., Collison, K. L., Lynam, D. R., & Miller, J. D. (2021). Does Machiavellianism meaningfully differ from psychopathy? It depends. *Behavioral Sciences & the Law*, *39*(5), 663–677. https://doi.org/10.1002/bsl.2538

Stanley, J. H., Wygant, D. B., & Sellbom, M. (2013). Elaborating on the Construct Validity of the Triarchic Psychopathy Measure in a Criminal Offender Sample. *Journal of Personality Assessment*, *95*(4), 343–350. https://doi.org/10.1080/00223891.2012.735302

Stanton, K., Brown, M. F. D., & Watson, D. (2021). Examining the Item-Level Structure of the Triarchic Psychopathy Measure: Sharpening Assessment of Psychopathy Constructs. *Assessment*, *28*(2), 429–445. https://doi.org/10.1177/1073191120927786

van Dongen, J. D. M., Drislane, L. E., Nijman, H., Soe-Agnie, S. E., & van Marle, H. J. C. (2017). Further Evidence for Reliability and Validity of the Triarchic Psychopathy Measure in a Forensic Sample and a Community Sample. *Journal of Psychopathology and Behavioral Assessment*, *39*(1), 58–66. https://doi.org/10.1007/s10862-016-9567-5

Venables, N. C., Hall, J. R., & Patrick, C. J. (2014). Differentiating psychopathy from antisocial personality disorder: A triarchic model perspective. *Psychological Medicine*, *44*(5), 1005–1013. https://doi.org/10.1017/S003329171300161X

Supplementary Table 3

Meta-analytically derived relations between sexual aggression and psychopathy at the triarchic level: Raw relative weights

|  | k | N | SE | Effect | σ |
| --- | --- | --- | --- | --- | --- |
| General sexual  aggression | | | | | |
| Boldness | 161 | 45145 | .10 | -.25** | .28 |
| Meanness | 161 | 45145 | .11 | -.05 | .28 |
| Disinhibition | 161 | 45145 | .25 | .35 | .28 |
| General sexual Aggression no CM | | | | | |
| Boldness | 141 | 43828 | .10 | -.25* | .25 |
| Meanness | 141 | 43828 | .11 | -.07 | .24 |
| Disinhibition | 141 | 43828 | .24 | .35 | .25 |
| Child molestation | | | | | |
| Boldness | 20 | 1317 | .92 | .29 | .26 |
| Meanness | 20 | 1317 | 1.05 | -1.41 | .24 |
| Disinhibition | 20 | 1317 | 1.02 | -1.63 | .24 |
| Undifferentiated | | | | | |
| Boldness | 104 | 37755 | .33 | .32 | .26 |
| Meanness | 104 | 37755 | .14 | .22 | .26 |
| Disinhibition | 104 | 37755 | .26 | .40 | .27 |

*Note*. ***p<.001, **p<.01, *p<.05; k = number of effect sizes.

Supplementary Table 4

Meta-analytically derived relations between sexual aggression and psychopathy: Remaining moderation results for sexual aggression forms with k < 20

|  | k | N | Reference Group | SE | Effect | σ |
| --- | --- | --- | --- | --- | --- | --- |
| Rape | | | | | | |
| How effect size produced | 8 | 762 |  |  |  | .32 |
| Compared groups |  |  | Direct correlation | .44 | .05 |  |
| Gender^b^ | -- | -- | -- | -- | -- | -- |
| Race | 7 | 720 |  | .00 | -.01 | .00 |
| Age | 7 | 720 |  | .01 | .00 | .18 |
| Sample type | 8 | 762 |  |  |  | .35 |
| Student |  |  | Sex Offenders | .52 | .02 | -- |
| Forensic non-SOs |  |  | Sex Offenders | .31 | .09 | -- |
| Country | 8 | 762 |  |  |  | .36 |
| Canada |  |  | United States | .29 | -.05 | -- |
| Italy |  |  | United States | .45 | -.00 | -- |
| SA measure source | 8 | 762 |  |  |  | .32 |
| Record review |  |  | Self-report | .44 | .05 | -- |
| SA measure | 8 | 762 |  |  |  | .32 |
| SES |  |  | Legal records | .44 | -.05 | -- |
| SA measure had non-SA items vs.  Not^b^ |  |  | No non-SA items | -- | -- | -- |
| Psy measure source | 8 | 762 |  |  |  | .19 |
| Self-report |  |  | Record review | .40 | -.58 | -- |
| Interview |  |  | Record review | .34 | -.53 | -- |
| Both interview & record review |  |  | Record review | .23 | -.66** | -- |
| Psy measure record review vs. not |  |  | Record review | .18 | -.64*** | .14 |
| Psy measure | 8 | 762 |  |  |  | .32 |
| SRP |  |  | PCL | .44 | -.05 | -- |
| Sex offender sample type^b^ | -- | -- | -- | -- | -- | -- |
| Victim class^b^ | -- | -- | -- | -- | -- | -- |
| Exhibitionism | | | | | | |
| How effect size produced | 3 | 317 |  |  |  | .00 |
| Compared groups |  |  | Direct correlation | .21 | .40 |  |
| Gender^b^ | -- | -- | -- | -- | -- | -- |
| Race^b^ | -- | -- | -- | -- | -- | -- |
| Age | 3 | 317 |  | .01 | .01 | .22 |
| Sample type | 3 | 317 |  |  |  | .24 |
| Student |  |  | Sex Offenders | .33 | -.16 | -- |
| Country | 3 | 317 |  |  |  | .24 |
| Canada |  |  | United States | .31 | .17 | -- |
| SA measure source | 3 | 317 |  |  |  | .00 |
| Record review |  |  | Self-report | .23 | .40 | -- |
| Combination |  |  | Self-report | .13 | -.00 |  |
| SA measure | 3 | 317 |  |  |  | .24 |
| Clinician ratings/DSM criteria |  |  | MASA | .33 | .16 | -- |
| SA risk assessment vs. not^b^ |  |  | -- | -- | -- | -- |
| SA measure had non-SA items vs.  not^b^ |  |  | -- | -- | -- | -- |
| Psy measure source | 3 | 317 |  |  |  | .24 |
| Both interview & record review |  |  | Self-report | .33 | .16 | -- |
| Psy measure record review vs. not^b^ |  |  | -- | -- | -- | -- |
| Psy measure | 3 | 317 |  |  |  | .24 |
| SRP |  |  | PCL | .33 | -.16 | -- |
| PCL vs. not^b^ |  |  | -- | -- | -- | -- |
| Sex offender sample type^b^ | -- | -- | -- | -- | -- | -- |
| Victim class^b^ | -- | -- | -- | -- | -- | -- |
| Voyeurism | | | | | | |
| How effect size produced | 4 | 1314 |  |  |  | .15 |
| Compared groups |  |  | Direct correlation | .35 | -.01 |  |
| Gender | 4 | 1314 |  | .00 | -.00 | .00 |
| Race | 3 | 1304 |  | .01 | -.01 | .03 |
| Age | 4 | 1314 |  | .01 | -.00 | .15 |
| Sample type | 4 | 1314 |  |  |  | .15 |
| Student |  |  | Sex Offenders | .35 | .01 | -- |
| Country^a^ | 4 | 1314 |  |  |  | .20 |
| Canada |  |  | United States | .10 | -.22 | -- |
| SA measure source | 4 | 1314 |  |  |  | .15 |
| Record review |  |  | Self-report | .35 | -.01 | -- |
| SA measure self-report vs. not^b^ | -- | -- | -- | -- | -- | -- |
| SA measure^a^ | 4 | 1314 |  |  |  | .03 |
| Clinician ratings/DSM criteria |  |  | MASA | .32 | .13 | -- |
| Freund's Paraphilia Scales |  |  | MASA | .11 | .23 | -- |
| SA risk assessment vs. not^b^ |  |  | -- | -- | -- | -- |
| SA measure had non-SA items vs.  not |  |  | -- | -- | -- | -- |
| Psy measure source | 4 | 1314 |  |  |  | .15 |
| Both interview & record review |  |  | Self-report | .35 | -.01 | -- |
| Psy measure record review vs. not^b^ |  |  | Record review | -- | -- | -- |
| Psy measure | 4 | 1314 |  |  |  | .00 |
| LSRP |  |  | PCL | .30 | .16 | -- |
| SRP |  |  | PCL | .31 | -.13 | -- |
| TriPM |  |  | PCL | .30 | .04 | -- |
| PCL vs. not |  |  | PCL | .35 | .01 | .15 |
| Sex offender sample type^b^ | -- | -- | -- | -- | -- | -- |
| Victim class^b^ | -- | -- | -- | -- | -- | -- |
| Mixed | | | | | | |
| How effect size produced |  |  |  |  |  |  |
| Compared groups^b^ | -- | -- | -- | -- | -- | -- |
| Gender^b^ | -- | -- | -- | -- | -- | -- |
| Race^b^ | -- | -- | -- | -- | -- | -- |
| Age | 4 | 107 |  | .02 | .00 | .08 |
| Sample type | 4 | 107 |  |  |  | .03 |
| Forensic non-SOs |  |  | Sex Offenders | .10 | -.04 | -- |
| Country | 4 | 107 |  |  |  | .08 |
| Canada |  |  | United States | .24 | .02 | -- |
| SA measure source^b^ | -- | -- | -- | -- | -- | -- |
| SA measure^b^ | -- | -- | -- | -- | -- | -- |
| Psy measure source^b^ | -- | -- | -- | -- | -- | -- |
| Psy measure^b^ | -- | -- | -- | -- | -- | -- |
| Sex offender sample type^b^ | -- | -- | -- | -- | -- | -- |
| Victim class^b^ | -- | -- | -- | -- | -- | -- |
| Sexual Homicide | | | | | | |
| How effect size produced | 3 | 262 |  |  |  | .00 |
| Compared groups |  |  | Direct correlation | .22 | .09 |  |
| Gender^b^ | -- | -- |  | -- | -- | -- |
| Race^b^ | -- | -- |  | -- | -- | -- |
| Age | 3 | 262 |  | .01 | .00 | .00 |
| Sample type | 3 | 262 |  |  |  | .00 |
| Forensic non-SOs |  |  | Sex Offenders | .12 | .02 | -- |
| Country | 3 | 262 |  |  |  | .00 |
| France |  |  | Canada | .12 | -.04 | -- |
| Finland |  |  | Canada | .23 | .07 | -- |
| SA measure source^b^ | -- | -- | -- | -- | -- | -- |
| SA measure^b^ | -- | -- | -- | -- | -- | -- |
| Psy measure source | 3 | 262 |  |  |  | .00 |
| Both interview & record review |  |  | Record review | .12 | .02 | -- |
| Psy measure^b^ | -- | -- | -- | -- | -- | -- |
| Sex offender sample type^b^ | -- | -- | -- | -- | -- | -- |
| Victim class^b^ | -- | -- | -- | -- | -- | -- |
| Sexual Cyberbullying/  harassment | | | | | | |
| How effect size produced^b^ | -- | -- | -- | -- | -- | -- |
| Gender^b^ | 2 | 1096 |  | .00 | -.02*** | -- |
| Race^b^ | -- | -- |  | -- | -- | -- |
| Age^b^ | -- | -- |  | -- | -- | -- |
| Sample type | 3 | 1470 |  |  |  | .31 |
| Community |  |  | Student | .38 | -.15 | -- |
| Country | 3 | 1470 |  |  |  | .00 |
| Germany |  |  | Australia | .06 | .44*** | -- |
| Spain |  |  | Australia | .07 | .37*** | -- |
| SA measure source^b^ | -- | -- |  | -- | -- | -- |
| SA measure | 3 | 1470 |  |  |  | .04 |
| Items measuring sexual  cyberbullying/harassment |  |  | Explicit Image Scale | .08 | .41*** |  |
| SA risk assessment vs. not^b^ |  |  | -- | -- | -- | -- |
| SA measure had non-SA items vs.  Not^b^ |  |  | -- | -- | -- | -- |
| Psy measure source^b^ | -- | -- |  | -- | -- | -- |
| Psy measure | 3 | 1470 |  |  |  | .00 |
| Dirty Dozen |  |  | LSRP | .07 | .37*** | -- |
| Naughty Nine |  |  | LSRP | .06 | .44*** | -- |
| Sex offender sample type^b^ | -- | -- |  | -- | -- | -- |
| Victim class^b^ | -- | -- |  | -- | -- | -- |
| Sexual Sadism | | | | | | |
| How effect size produced | 14 | 3071 |  |  |  | .18 |
| Compared groups |  |  | Direct correlation | .15 | .32 |  |
| Gender | 14 | 3071 |  | .00 | -.00 | .23 |
| Race | 11 | 2714 |  | .00 | -.00 | .08 |
| Age | 14 | 3071 |  | .01 | .01 | .23 |
| Sample type | 14 | 3071 |  |  |  | .24 |
| Student |  |  | Sex Offenders | .20 | .03 | -- |
| Forensic non-SOs |  |  | Sex Offenders | .21 | -.08 | -- |
| Country | 14 | 3071 |  |  |  | .18 |
| Canada |  |  | United States | .16 | .33 | -- |
| France |  |  | United States | .22 | -.02 |  |
| Switzerland |  |  | United States | .21 | -.22 |  |
| UK |  |  | United States | .23 | .11 | -- |
| SA measure source | 14 | 3071 |  |  |  | .25 |
| Record review |  |  | Self-report | .06 | -.19 | -- |
| Clinician ratings |  |  | Self-report | .28 | -.18 | -- |
| Combination |  |  | Self-report | .22 | -.16 | -- |
| SA measure self-report vs. not | 14 | 3071 | Self-report | .06 | -.18** | .22 |
| SA measure | 14 | 3071 |  |  |  | .29 |
| Clinician ratings/DSM criteria |  |  | MASA | .33 | -.00 | -- |
| Freund's Paraphilia Scales |  |  | MASA | .42 | .14 | -- |
| MIDSA |  |  | MASA | .42 | .12 | -- |
| MTC |  |  | MASA | .42 | -.08 |  |
| SeSaS |  |  | MASA | .33 | .11 | -- |
| Sexual Sadism Criteria of  Offenses from Dietz et al. (1990) |  |  | MASA | .53 | -.27 | -- |
| SA risk assessment vs. not^b^ |  |  | -- | -- | -- | -- |
| SA measure had non-SA items vs.  not |  |  | No non-SA items | .05 | .12 | .20 |
| Psy measure source | 14 | 3071 |  |  |  | .22 |
| Self-report |  |  | Record review | .20 | .15 | -- |
| Both interview & record review |  |  | Record review | .18 | .25 | -- |
| Other combination |  |  | Record review | .28 | .18 | -- |
| Psy measure record review vs. not |  |  | Record review | .13 | .20 | .20 |
| Psy measure | 14 | 3071 |  |  |  | .24 |
| LSRP |  |  | PCL | .26 | .15 | -- |
| SRP |  |  | PCL | .27 | -.03 |  |
| TriPM |  |  | PCL | .26 | .07 |  |
| Sex offender sample type | 11 | 1767 |  |  |  | .28 |
| Undifferentiated SOs |  |  | SSSOs | .10 | .12 | -- |
| Sexual homicide offenders |  |  | SSSOs | .24 | .04 | -- |
| Victim class^b^ | -- | -- | -- | -- | -- | -- |

*Note*. ***p<.001, **p<.01; p<.05 is not signified because a p<.01 significance level was used for these moderation analyses. k = number of effect sizes. SA = sexual aggression. SOs = sexual offenders; SSSOs = sexually sadistic sexual offenders; DSM = Diagnostic and Statistical Manual of Mental Disorders; MASA = Multidimensional Assessment of Sexual Aggression; MIDSA = Multidimensional Inventory of Development, Sex, and Aggression; MTC = Massachusetts Treatment Center; SeSaS = Severe Sexual Sadism Scale; SRP = Self-Report Psychopathy Scale; PCL = Psychopathy Checklist; LSRP = Levenson Self-Report Psychopathy scale; TriPM = Triarchic Psychopathy Measure. a. Given the numerous levels of this categorical moderator and space constraints, only results for levels that were significantly different from the reference group are included here. b. This analysis could not be conducted due to too few studies, too much missing data for moderator variable, and/or too few levels of a categorical moderator.

*Supplementary Figure 1.* PRISMA Flowchart

Studies from databases/registers **(n = 2314)**

PubMed (n = 1223)

PsycINFO (n = 1091)

**Identification**

References removed **(n = 274)**

Duplicates identified manually (n = 7)

Duplicates identified by Covidence (n = 267)

­

Studies screened **(n = 2040)**

Studies excluded **(n = 1626)**

Studies excluded **(n = 327)**

Not empirical (n = 10)

Not in English (n = 15)

Duplicate sample (n = 88)

Not adult sample (n = 1)

Synthesized data (n = 1)

Duplicate article (n = 8)

No comparison group (n = 88)

No psychopathy measure (n = 43)

Could not get full text (n = 8)

No sexual aggression measure (n = 37)

Could not get effect size from authors (n = 23)

No psychopathy nor sexual aggression

measure (n = 5)

Used only behavioral task for psychopathy or sexual aggression (n = 1)

Studies assessed for eligibility **(n = 414)**

**Screening**

Eligible studies **(n = 87)**

Unpublished studies acquired from researchers **(n = 4)**

Studies found from ancestry search **(n = 4)**

Total studies included **(n = 95)**

**Included**

*Supplementary Figure 2.* Funnel plots characterizing effect sizes between global psychopathy and sexual aggression


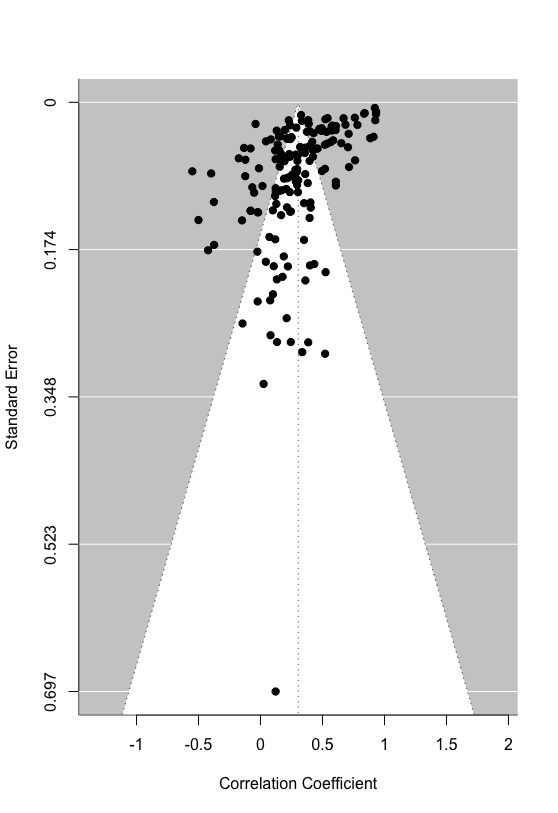


Global psychopathy and general sexual aggression


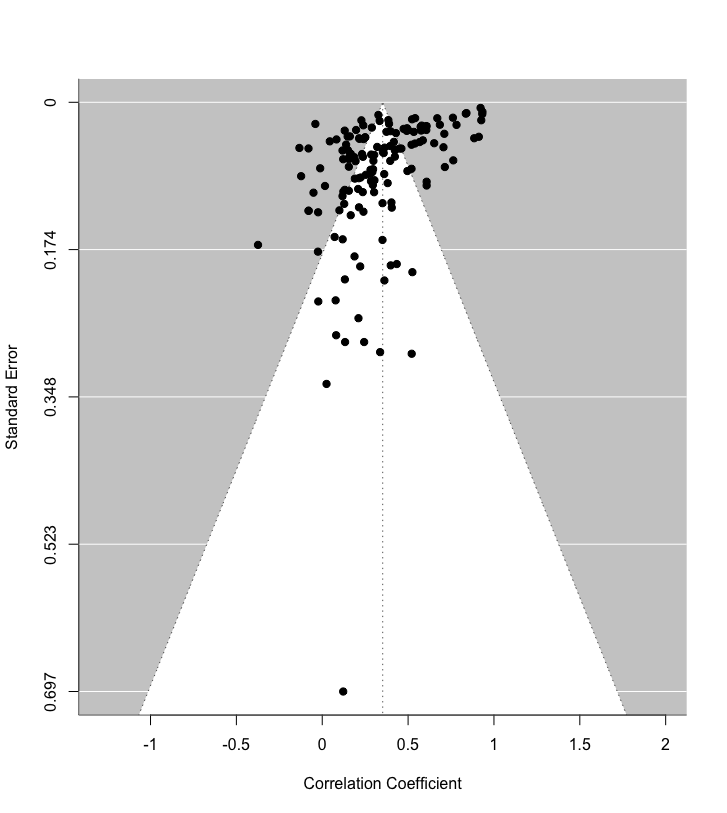


Global psychopathy and general sexual aggression without child molestation


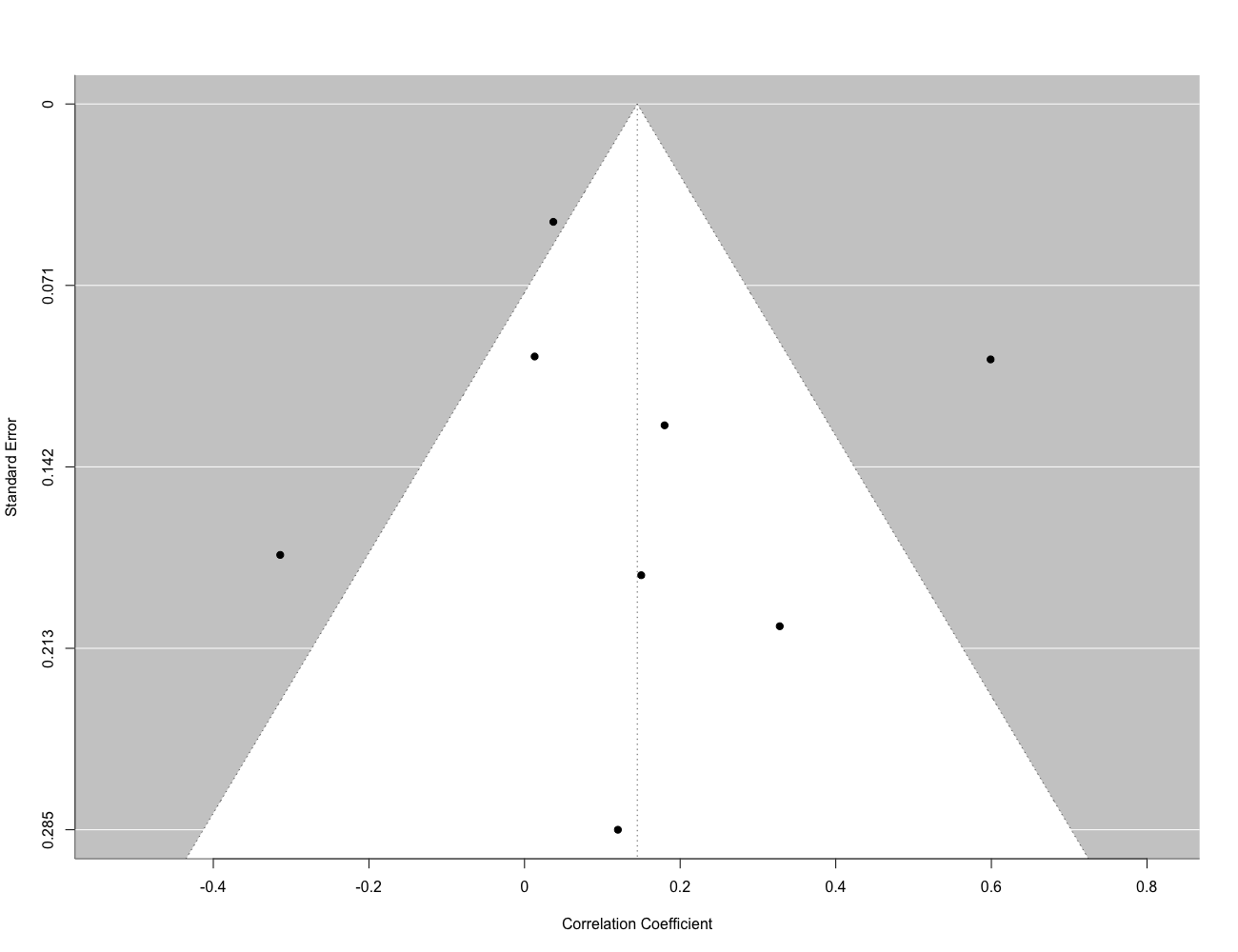


Global psychopathy and rape


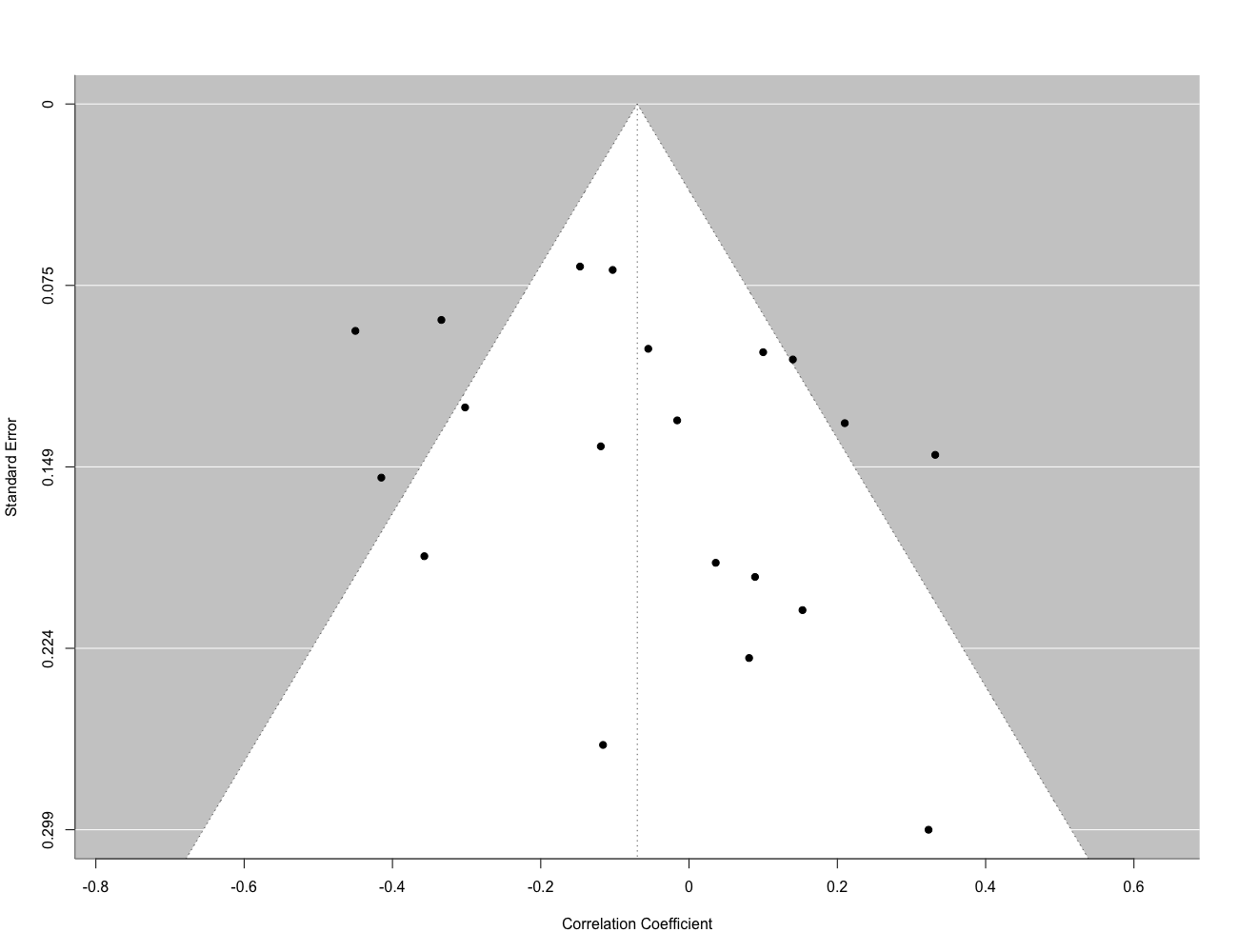


Global psychopathy and child molestation


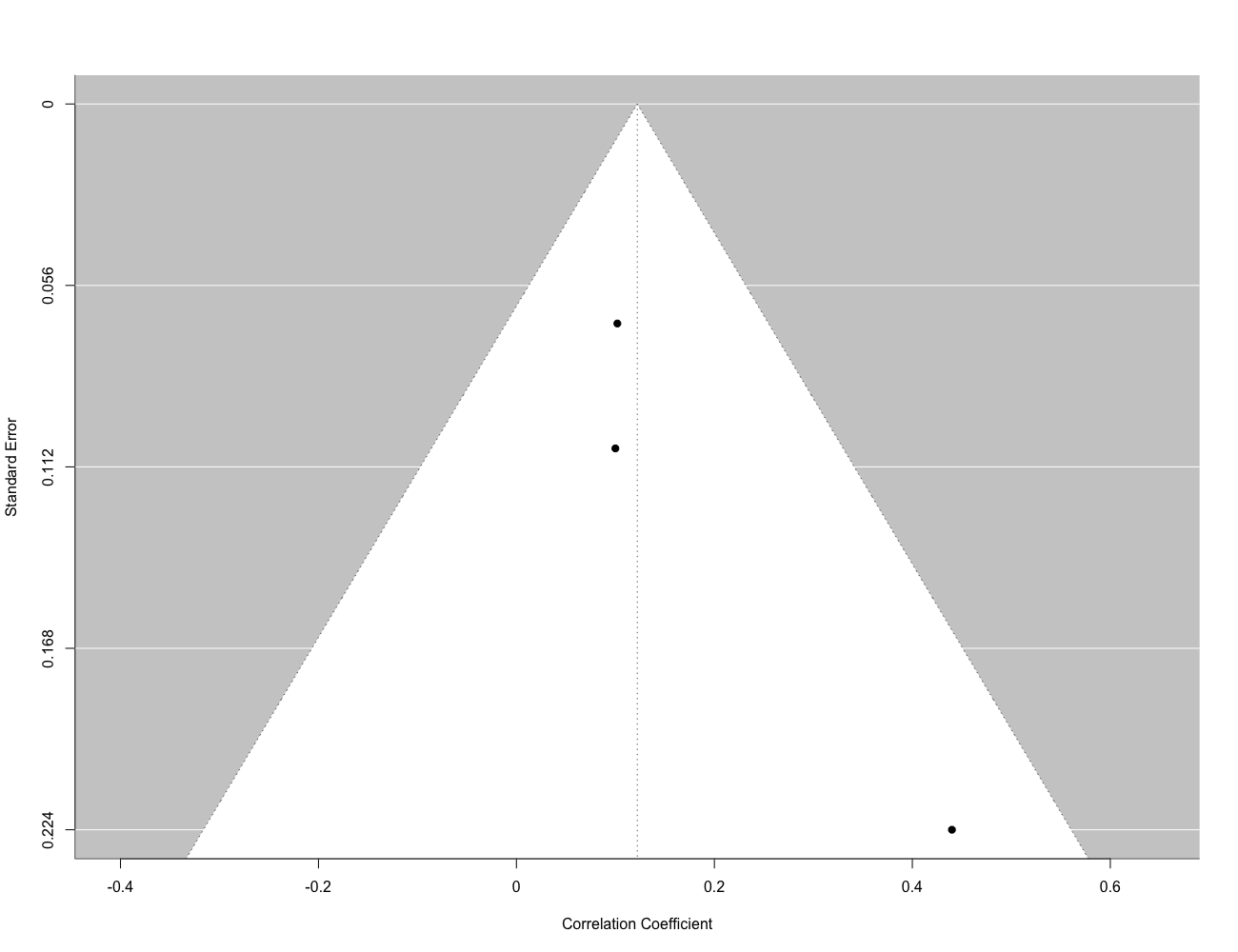


Global psychopathy and exhibitionism


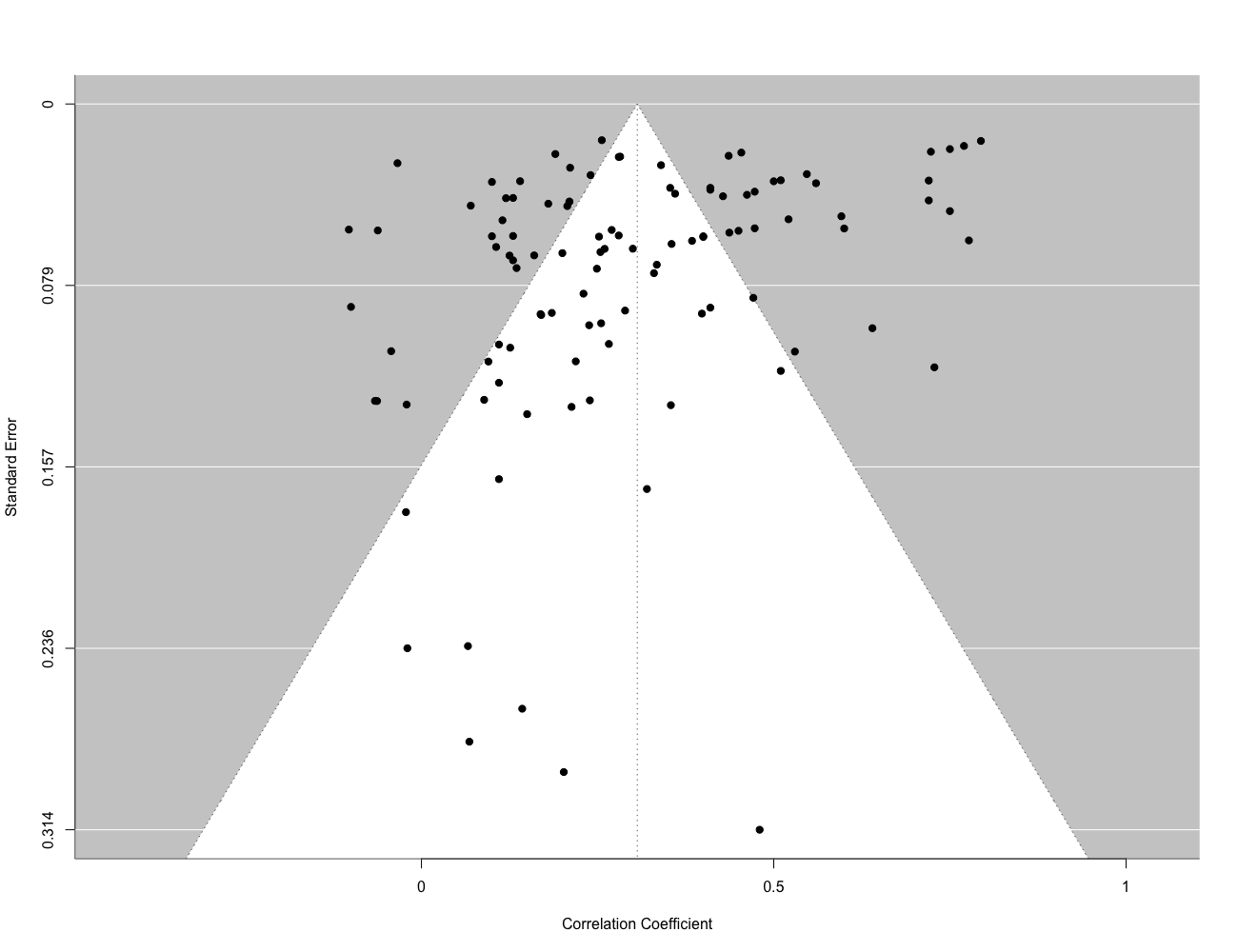


Global psychopathy and undifferentiated sexual aggression


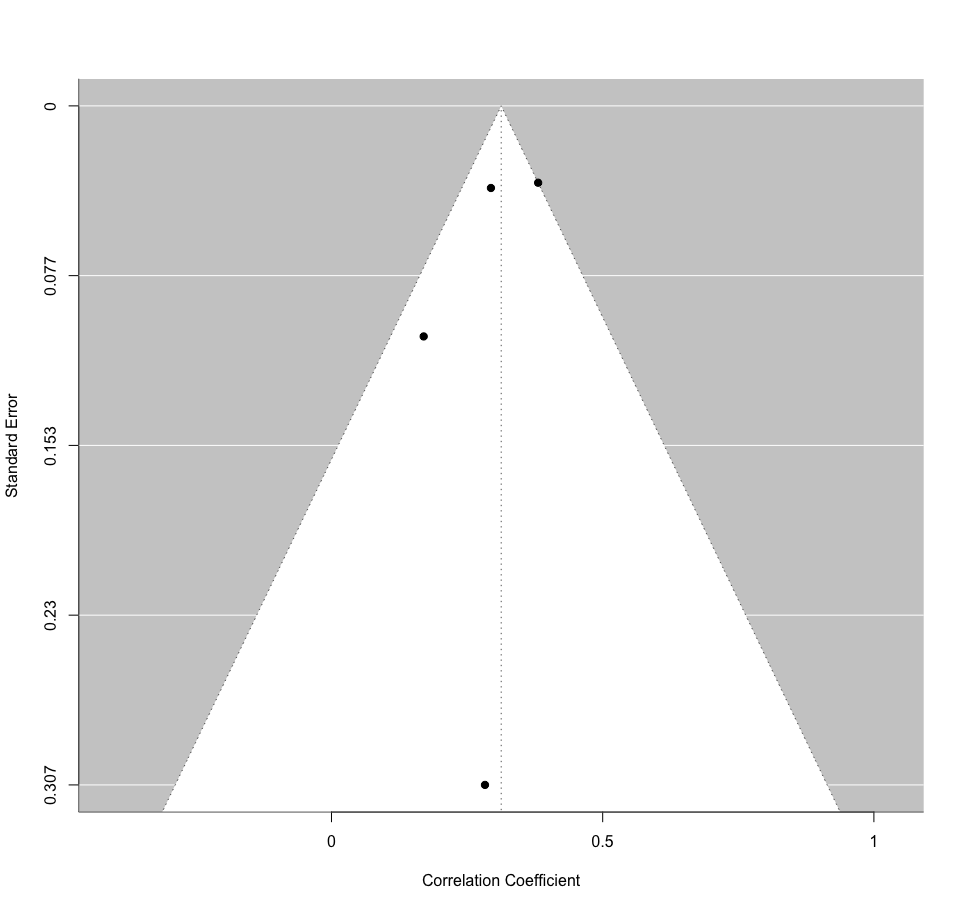


Global psychopathy and voyeurism


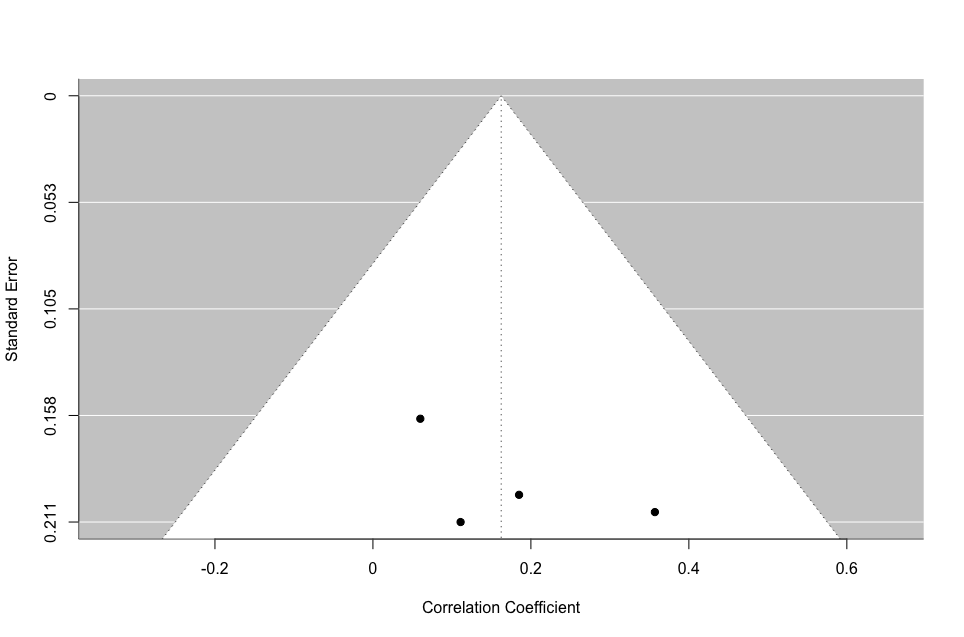


Global psychopathy and mixed sexual aggression


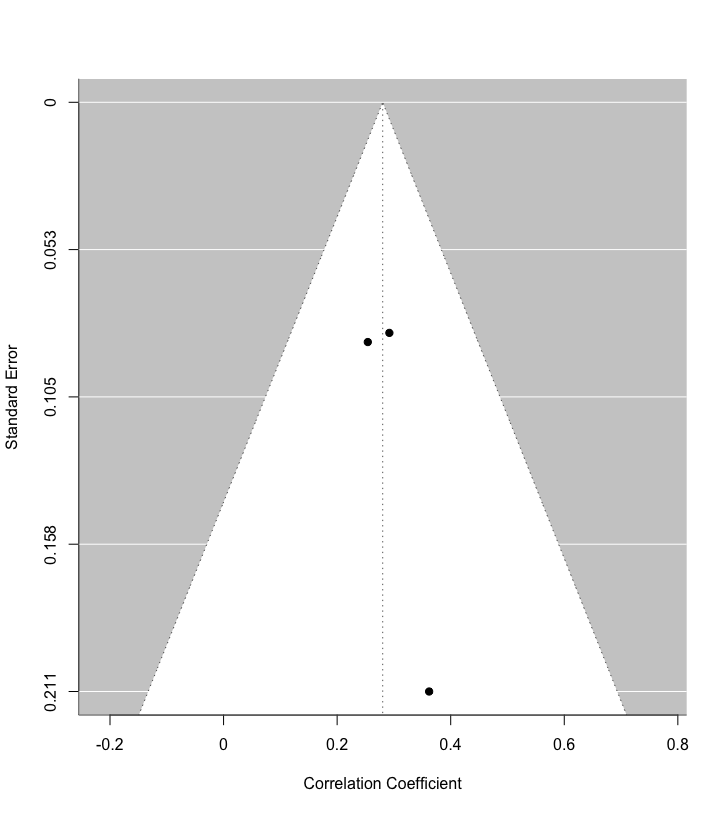


Global psychopathy and sexual homicide


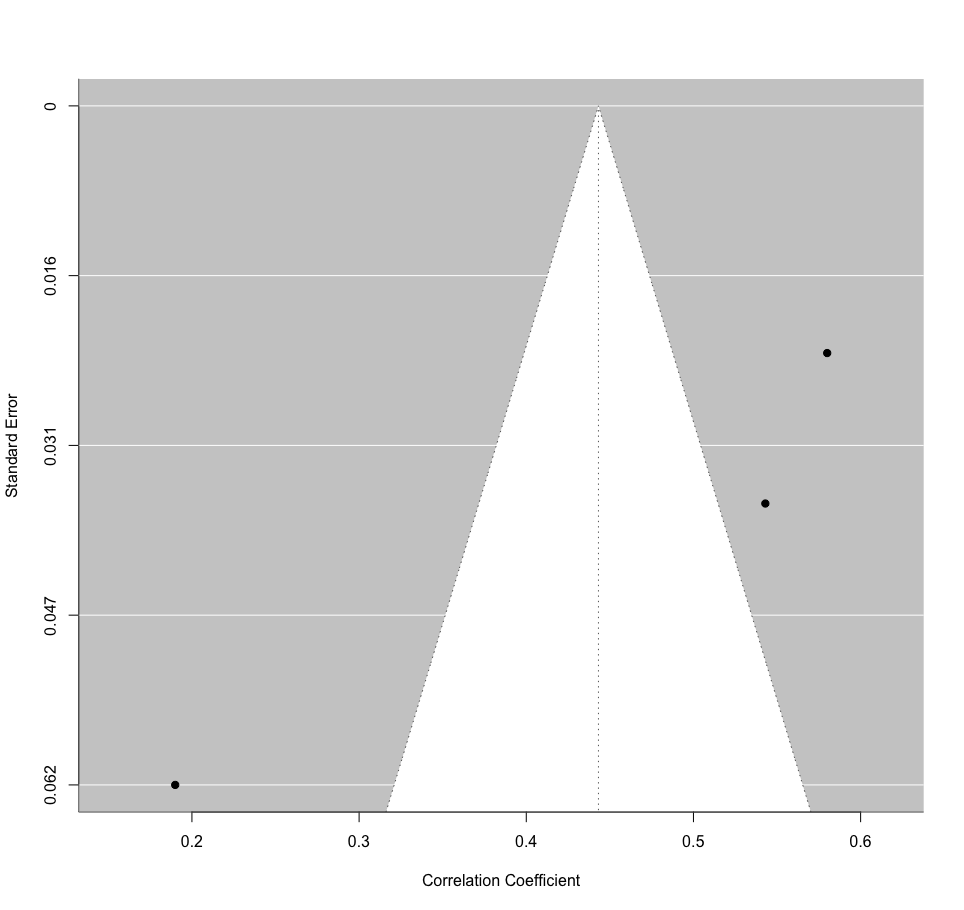


Global psychopathy and sexual cyberbullying/harassment


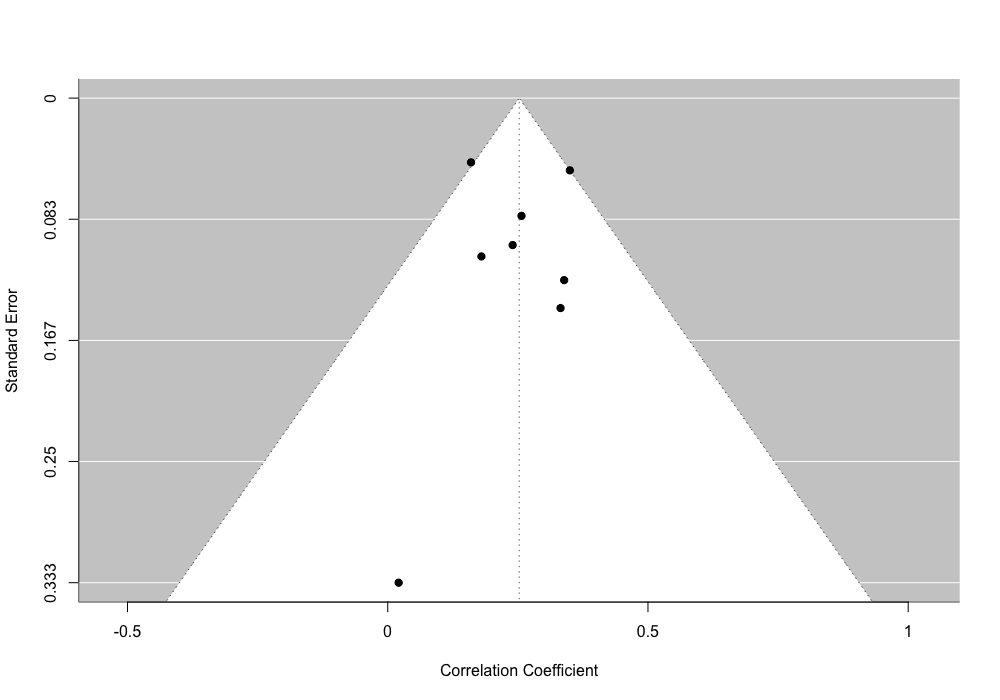


Global psychopathy and sexual sadism
